# Metabolic Reprogramming of Thyroid Cancer Cells and Crosstalk in Their Microenvironment

**DOI:** 10.3389/fonc.2021.773028

**Published:** 2021-12-02

**Authors:** Lisha Bao, Tong Xu, Xixuan Lu, Ping Huang, Zongfu Pan, Minghua Ge

**Affiliations:** ^1^ Second Clinical College, Zhejiang Chinese Medical School, Hangzhou, China; ^2^ ENT-Head & Neck Surgery Center, Department of Head and Neck Surgery, Zhejiang Provincial People’s Hospital, Affiliated People’s Hospital, Hangzhou Medical College, Hangzhou, China; ^3^ Key Laboratory of Endocrine Gland Diseases of Zhejiang Province, Zhejiang Provincial People’s Hospital, Hangzhou, China; ^4^ Clinical Pharmacy Center, Department of Pharmacy, Zhejiang Provincial People’s Hospital, Affiliated People’s Hospital, Hangzhou Medical College, Hangzhou, China

**Keywords:** metabolic reprogramming, thyroid cancer, microenvironment, metabolic interplay, Warburg effect

## Abstract

Metabolism differs significantly between tumor and normal cells. Metabolic reprogramming in cancer cells and metabolic interplay in the tumor microenvironment (TME) are important for tumor formation and progression. Tumor cells show changes in both catabolism and anabolism. Altered aerobic glycolysis, known as the Warburg effect, is a well-recognized characteristic of tumor cell energy metabolism. Compared with normal cells, tumor cells consume more glucose and glutamine. The enhanced anabolism in tumor cells includes *de novo* lipid synthesis as well as protein and nucleic acid synthesis. Although these forms of energy supply are uneconomical, they are required for the functioning of cancer cells, including those in thyroid cancer (TC). Increasing attention has recently focused on alterations of the TME. Understanding the metabolic changes governing the intricate relationship between TC cells and the TME may provide novel ideas for the treatment of TC.

## Introduction

Thyroid cancer (TC) remains the most frequently diagnosed endocrine malignancy; with a sharp increase in incidence worldwide, this disease is projected to become the fourth leading type of cancer globally (1). Based on its histological features, TC is grouped into four types: papillary thyroid carcinoma (PTC), follicular thyroid carcinoma (FTC), medullary thyroid cancer (MTC), and anaplastic thyroid carcinoma (ATC). Approximately 90% of all TCs are differentiated, including PTC, which is the most common histological type of differentiated thyroid cancer, followed by FTC (2). Notably, different TC subtypes exhibit distinct tumor aggressiveness and progression and show heterogeneous responses to different treatments (3). Although well-differentiated TCs have good prognoses, approximately 10% of patients do not respond to radioactive iodine therapy and are more likely to relapse. While the incidence of poorly differentiated TCs such as ATC and MTC is very low, they are characterized by high invasiveness, early metastasis, and poor prognosis (4, 5). Conventional therapy consists of surgery, radiotherapy, and endocrine suppression treatment (6, 7). However, these treatments have various limitations and side effects (8, 9).

The large differences in metabolism between tumor cells and normal human somatic cells are mainly reflected in catabolic and biosynthesis metabolism (10). The metabolic changes in tumor cells are often considered to be closely related to tumor formation and progression (11). Thus, the unique metabolism of tumor cells is both an opportunity and a challenge. Here, we review the catabolic and anabolic metabolism changes in TC cells. We also describe the mutual relationship between metabolic reprogramming and the tumor microenvironment (TME) in TC, which provides the theoretical basis for new therapeutic targets and prognostic indicators.

## Metabolic Changes in Tumor Cells

Cancer cells always acquire energy and material basis for rapid tumor growth by enhanced anabolism, including rapid aerobic glycolysis, glutaminolysis, *de novo* lipid synthesis and nucleotide synthesis (12, 13). Thyroid cancer cells generate energy primarily by increasing glycolysis and glutaminolysis. In addition, the production of glycolysis can also provide materials for nucleic acid synthesis through pentose phosphate pathway (PPP). Nucleic acid synthesis, protein synthesis, and *de novo* lipid synthesis are enhanced to support thyroid cancer cell proliferation. During metastasis, tumor cells rely on catabolism to survive from metabolic stress, mainly through aerobic glycolysis, OXPHOS, glutamine metabolism and autophagy to produce ATP (14). Thyroid tumors acquired aggressive phenotype and epithelial-mesenchymal transformation(EMT) *via* sirtuin 6 (SIRT6)-Autophagy-Warburg Effect Axis (15). AMPK signal is also essential for activating adaptive changes in cell metabolism such as inhibiting anabolism and promoting catabolism, which is the basis for cell survival under metabolic stress. In TC, AMPK activation inhibits TC cell proliferation and promotes cell migration (16). Moreover, carnitine palmitoyltransferase 1C which is regulated by AMPK, transfers long-chain fatty acids into mitochondria to further oxidation and promotes TC cells survival under metabolic stress conditions (17).

### Changes in Catabolism

#### Glucose Metabolism

**Figure 1:**
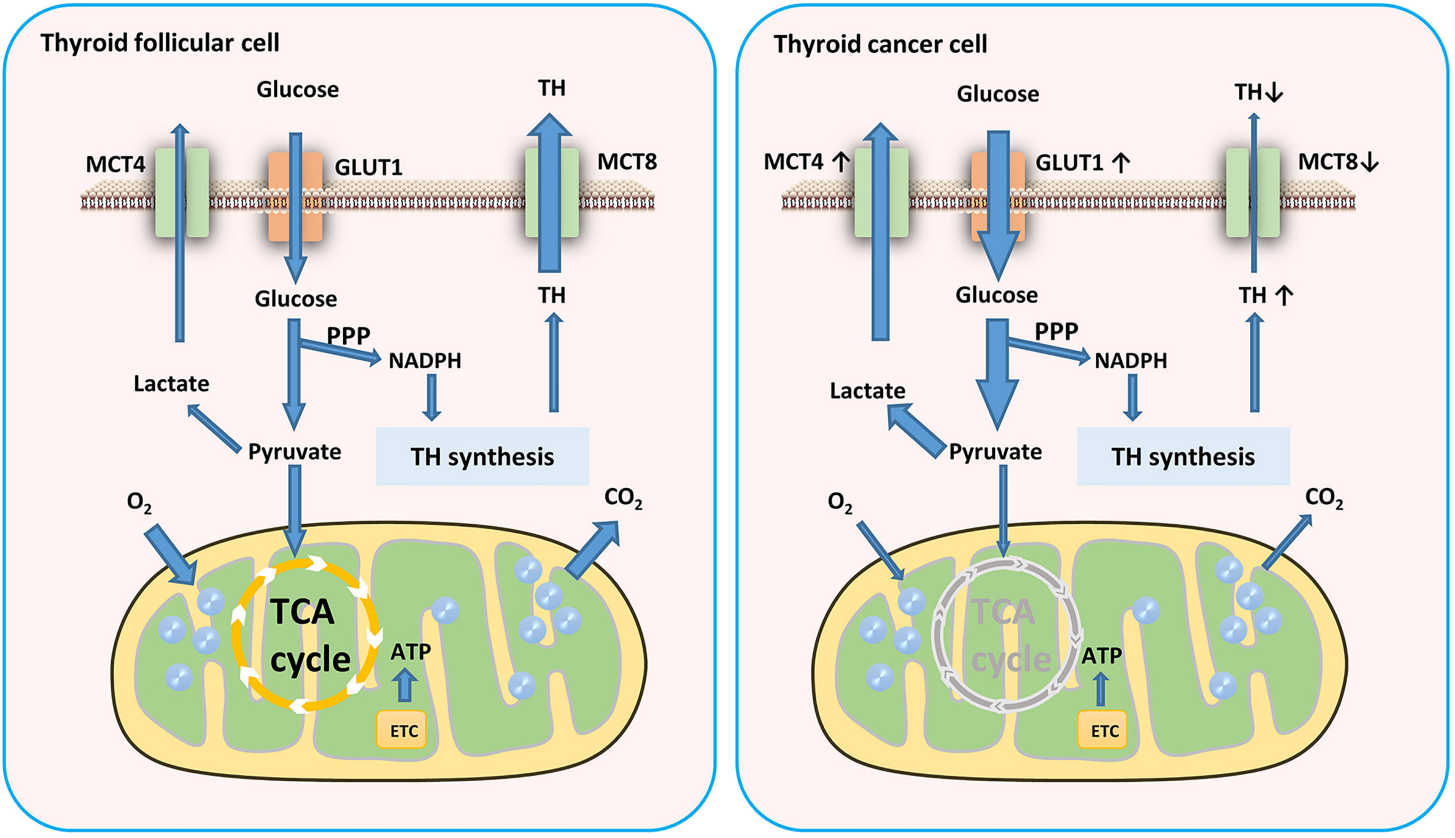
Glucose metabolism in TC cells. TC cells require more glucose than normal cells and derive their energy mainly from glycolysis. This aerobic glycolytic phenotype generates more lactates which transported by MCT4. MCT8 downregulation in TC cells results in TH accumulation in TC tissues. GLUT, glucose transporter; TH, thyroid hormones; ETC, electron transport chain; MCT, monocarboxylate transporter.

Cells produce ATP for energy in two main ways: glycolysis and oxidative phosphorylation (OXPHOS). To satisfy the need of energy for proliferation, thyroid tumor cells increased the level of glycolysis. Although aerobic glycolysis is inefficient compared to OXPHOS, it can provide energy for tumor cell proliferation and invasion and a constant supply of material for biosynthesis (18). The Warburg effect suggests that tumor cells require more glucose than normal cells and derive their energy mainly from glycolysis even when oxygenated adequately (19). However, the energy sources of different tumors also show heterogeneity, and even different areas of the same tumor have different energy sources (20–22). It is noteworthy that glycolysis plays a more important role in sustaining the balance of the PPP in thyroid cells, which is more critical for thyroid hormone synthesis than ATP production even in TC (23) (**Figure 1**).

Hypoxia-inducible factor (HIF) is a transcription factor that is widespread in mammals and humans under hypoxic conditions. HIF plays roles in glycolysis, promote angiogenesis, cell survival or apoptosis. As the basic regulator of glycolysis, HIF can upregulate the activity of 90% of glycolytic reactivity enzymes and inhibit the use of pyruvate by mitochondria (24). In TC cells, aerobic glycolysis can be enhanced through the alteration of the HIF1α-MYC-PGC-1β axis (25). Zhou et al. showed that hypoxia promoted FTC progression by upregulating HIF1α and programmed death-ligand 1 (PD-L1) (26). In PTC, SIRT6 promotes the EMT of cancer cells through HIF-1α (27). Klaus et al. demonstrated the critical role of HIF-1α in the desmoplastic stroma reaction and metastatic processes in FTC (28). HIF can stimulate the expression of MYC, a transcription factor that is highly expressed in tumors and has a variety of biological functions, including cell metabolism. *MYC* can promote glycolysis and glucose transporter (GLUT) expression, thus transforming tumor energy metabolism into the Warburg effect (29–31). Myc overexpression can also lead to abnormally increased synthesis of lactate dehydrogenase A (LDHA), which catalyzes pyruvate to lactate. Compared to normal thyroid tissues, LDHA expression is higher in PTC. Hou et al. reported that LDHA not only promoted PTC tumorigenesis but also migration and invasion by regulating autophagy and inducing EMT gene transcription. Moreover, they also found that the metabolic products catalyzed by LDHA increased the acetylation of the related *H3K27* and induced EMT (32). LDHA is phosphorylated by HER2 and SRC39, resulting in the increased invasive and metastatic potential of head and neck cancer (33).

GLUT is a transporter that helps cells to take up glucose and is the first rate-limiting step in glucose metabolism. Many studies have demonstrated the upregulation of GLUT subtypes during carcinogenesis (34–36). Samih et al. reported that the phosphoinositide 3-kinase (PI3K)/Akt pathway is the key to GLUT1 transfer from the cytoplasm to the plasma membrane (37). GLUT1 overexpression is also associated with cancer cell aggressiveness and dedifferentiation. Mediated by the transcription factor HIF, GLUT3 is upregulated in response to hypoxia. The overexpression of GLUT1 and GLUT3 is generally recognized as one of the characteristics of tumors (38). Jóźwiak et al. reported that most PTC samples showed higher GLUT1 and GLUT3 expression than the expression in FTC and non-neoplastic thyroid lesions (39). Chai et al. analyzed the expression of GLUT family genes and concluded that the upregulation of the genes encoding GLUT1, GLUT3, GLUT14 was associated with decreased overall survival in patients with PTC (40).The function and tissue distribution of GLUT14 are uncharacterized, although there is some disease association, specifically in inflammatory bowel disease. GLUT14 is a GLUT3 variant that has also been found in the genome as a duplicon of GLUT3. Moreover, the upregulation of GLUT14 was associated with the maintenance of glucose uptake in hypoxia (41). The localization of GLUT1 is heterogeneous among TCs. For example, it exhibits a focal circumferential form in plasma membrane of PTC cells, shows a non-symmetric distribution in the basilar membrane of tumor cells adjacent to the capillary blood supply and stroma, and focal distribution in the center of metastatic tumors or ATC (42). Previous studies indicated that GLUT1 and GLUT3 expression levels may be associated with increased invasion and a worse prognosis of TC. Glucose transported by GLUT involved in glycolysis, the products of which eventually enter the mitochondria to generate ATP for cell energy through OXPHOS. The mitochondrial pyruvate carrier 1 (MPC1) is a critical channel that connects glycolysis to OXPHOS by regulating the transport of pyruvate into the mitochondrial inner membrane. MPC1 deficiency may cause metabolic reprogramming and is associated with a poor prognosis. MPC1 expression is strongly negatively correlated with tumor purity and immune cell infiltration in TC (43).

Many enzymes are involved in the aerobic glycolysis of tumor cells, including pyruvate kinase M2 (PKM2), hexokinase (HK), phosphofructokinase 1 (PKF1). The PI3K/Akt pathway can enhance the Warburg effect of tumors by increasing the activity of these factors (44). HK is the first rate-limiting enzyme in glycolysis and catalyzes the phosphorylation of glucose into glucose 6-phosphate. HK2 is also highly expressed in TC (45, 46). Huang et al. demonstrated the promotion of thyroid carcinoma cell proliferation and migration through the activation of AKT/mTOR/HK2-mediated glycolysis (47). Feng et al. reported that PKM2 overexpression in PTC was related to poor clinicopathological features such as advanced tumor stages and lymph node metastasis (48). In their proteomic analysis of five PTC specimens, Aurélie Strickaert et al. investigated the cellular distribution of several upregulated metabolic proteins in the cancerous and stromal cells of these tumors. They discovered the upregulation of many metabolism-related proteins including pyruvate carboxylase (PC) (49). Verhagen et al. compared PK in human thyroid carcinomas, follicular adenomas, and normal thyroid tissue and reported a positive correlation between the specific activities of PK and tumor proliferation (50). The results of these studies demonstrated that PK overexpression plays an important role in TC.

#### Amino Acid Metabolism

Glutamine is a nonessential amino acid in normal cells and can be converted from glucose. However, tumor cells cannot grow in a culture medium without glutamine; thus, glutamine is an essential amino acid in these cells (51). Ample evidence supports the essential role of glutamine in tumors. Tumor cells consume large amounts of glutamine as an alternative energy supply pathway to glycolysis (52–54). However, the requirements for glutamine in cancer vary in different tissues and situations (55) (**Table 1**) . Several studies demonstrated the changes in glutamine metabolism of thyroid tumors. Inhibition of glutamine metabolism in TC cells results in insufficient energy supply, which inhibits cell proliferation, migration, and invasion (56). Kim et al. performed tissue microarrays of 557 TC cases and immunohistochemical staining of glutaminolysis-related proteins. They reported that glutaminase 1 (GLS1) and glutamate dehydrogenase (GDH) showed the highest expression in ATC compared to other subtypes. Tumoral amino acid transporter-2 expression was higher in MTC but lower in FTC. In PTC, the expression levels of tumoral GLS1 and GDH were higher in the conventional type than those in the follicular variant, and in the BRAF^V600E^ mutation than those in cases without the BRAF^V600E^ mutation (57). The expression levels of glutaminolysis-related proteins including GLS1, GDH, and GLUD were higher in Hürthle cell neoplasm of the thyroid than in those of follicular neoplasm. The expression of SLC1A5 was highest in Hürthle cell adenomas, followed by FC and FA (58). When glutamine enters the cell, it is hydrolyzed to glutamic acid and ammonia by glutaminase. Glutamate can be converted into α-KG to enter the tricarboxylic acid (TCA) cycle, providing intermediate metabolites and energy for cell metabolism. This is particularly evident in the truncated TCA cycle, which can be used as feedstock for the passive TCA cycle due to the lack of citrate (44). This phenomenon, termed anapleurosis, suggests that the use of glutamine affects glucose absorption. Therefore, reducing the use of glutamine can also reduce that of glucose (59). In general, glucose and glutamine metabolism influence each other. Other changes in protein metabolism are present besides glutamine. Sun et al. analyzed 557 different types of TC and found a higher expression level of serine/glycine metabolism-related proteins in PDC and PTC compared to that in MTC. In PTC, the rate of expression was higher in cases with BRAF^V600E^ mutation than in those with a follicular variant (60).

**Table 1:** The metabolic differences and similarities in cancers.

Metabolic pathways	Tumor types	Difference	Similarity
**Glycolysis metabolism**	Thyroid cancer	Produce NAPDH through the PPP pathway for thyroid hormone synthesis, ATP production (17)	Enhancement of glycolysis and lactate production
	Other cancers	Mainly used for ATP production (13)	
**Energy source**	Primary thyroid cancer	Glucose and glutamine metabolism(186)	Increased energy demand
	Metastatic thyroid cancer	Unknown	
	Primary breast cancer	Glucose and glutamine metabolism (15)	
	Metastatic breast cancer	Pyruvate (lung metastases) to sustain the TCA cycle (15) Serine and acetate (brain metastases) to sustain the TCA cycle (16)	
	Non-small cell lung cancer	Carbon source: glucose (areas with low perfusion); glucose and other sources (highly perfused areas) (14)	
**Lipid metabolism**	Thyroid cancer	Low correlation between MUFAs and MUPCs or monosaturated and polyunsaturated lipids (85) ACC2 downregulation (83)	Enhancement of de novo lipid synthesis
	Breast, lung, colorectal, esophageal and gastric cancer	Highly positive correlation between MUFAs and MUPCs negative correlation between monosaturated and polyunsaturated lipids (85)	
	liver, breast and prostate	ACC upregulation (82)	

### Changes in Biosynthesis Metabolism

#### Enhancement of *De Novo* Lipid Synthesis

Compared to normal tissue, tumor cells synthesize lipids more rapidly and from different sources. Accumulating evidence has demonstrated the important role of lipid metabolism reprogramming in tumor cell development and metastasis (61–67). Liao et al. reported that lysine methyltransferase 5A (KMT5A), a regulator of lipid metabolism in PTC, was significantly associated with extrathyroidal extension and lymph node metastasis in PTC (68). Instead of nutrient uptake, the raw materials of lipid synthesis in tumor cells mainly come from glucose metabolism. Approximately 93% of the fatty acids in tumor cells are synthesized *de novo* (69, 70). The enzymes involved in the fatty acid synthesis, such as ATP citrate lyase (ACLY), Acetyl-CoA carboxylase (ACC), and fatty acid synthase (FASN) are changed in tumor cells (71–83). Citrate, the intermediate product of glucose metabolism, forms Ac-CoA under the catalysis of ACLY, and Ac-CoA forms malonyl CoA (Mal-CoA) under the catalysis of ACC. Ac-CoA and MAL-CoA synthesize palmitic acid catalyzed by FASN, and palmitic acid forms lipid components required by cells catalyzed by other specific enzymes.

**Figure 2:**
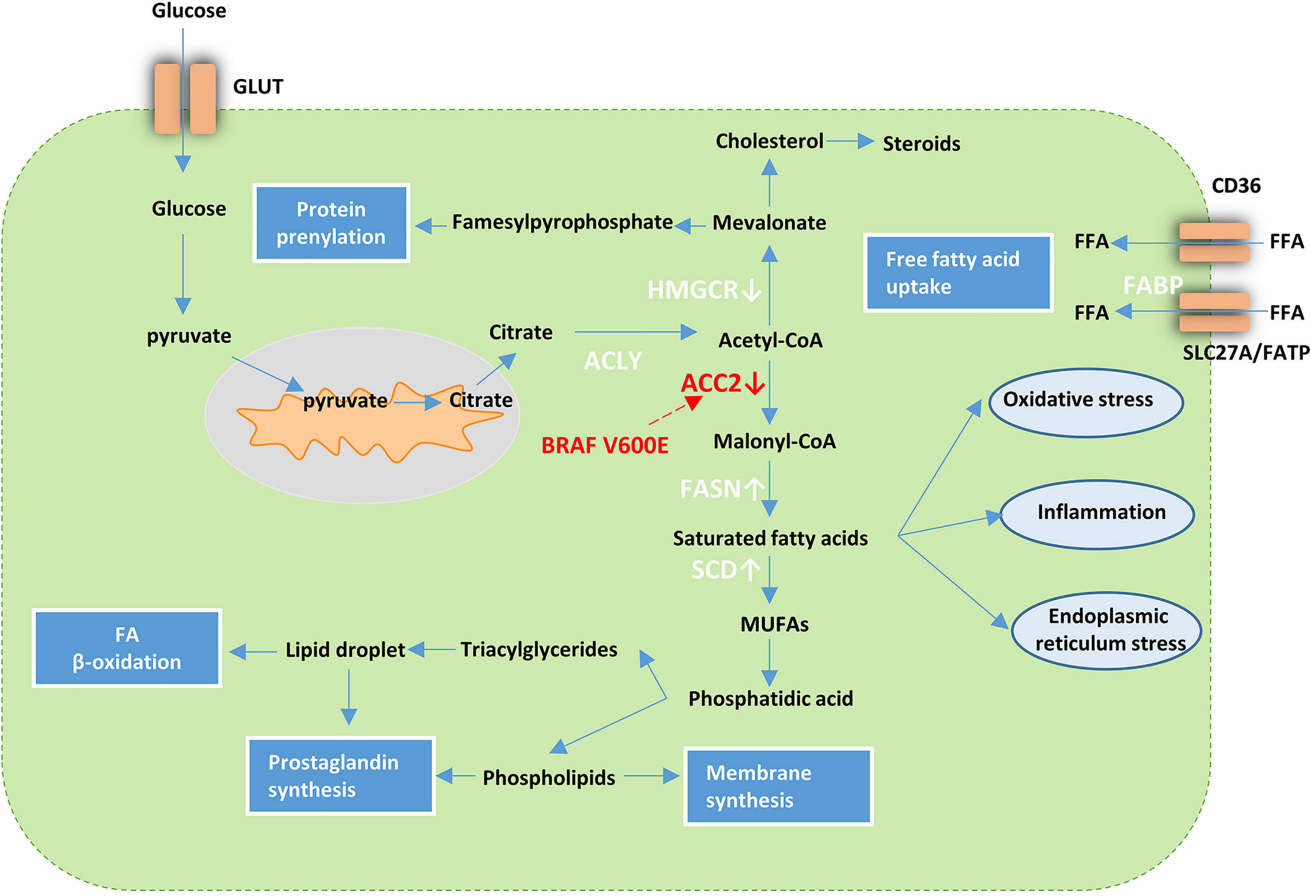
Lipid metabolism in cancer cells. Tumor cells increase FFA uptake *via* upregulation of fatty acid transport receptors and chaperones such as Solute Carrier SLC27A/FATP, CD36, and FABP. In addition, metabolic reprogramming that facilitates glycolysis can activate *de novo* lipid synthesis. Acetyl-CoA derived from citrate can be further processed into a variety of lipid species with the help of various enzymes. FASN and SCD are upregulated while ACC2 and HMGCR are downregulated in TC. BRAF^V600E^ influences the lipid metabolism in PTC *via* downregulation of ACC2. GLUT, glucose transporter; HMGCR, 3-hydroxy-3-methylglutaryl-CoA reductase; fatty acid synthase ACLY; ACC2, Acetyl-CoA carboxylase 2; FASN fatty acid synthase; SCD, stearoyl-CoA desaturase-1; MUFAs, monounsaturated fatty acids; FFA, free fatty acid; FABP, fatty acid binding protein; SLC27A, Solute Carrier Family 27; FATP, Fatty Acid Transporter.

Several studies on thyroid carcinoma also demonstrated lipid metabolism reprogramming. In their transcriptome analysis of lipid metabolism-related genes in PTC, Xu et al. described the use of these genes for PTC classification (84). Recent cases reported by Leng et al. suggested abnormality in the metabolism of fatty acid synthases and lipids. They detected 18 types of FFAs with increased levels in carcinoma tissue compared to the normal tissue of the thyroid (85). Several studies have reported abnormal changes in lipogenic enzymes in TC. FASN is upregulated in various TC subtypes, including PTC, ATC, and FTC (86–88). Under hypoxic conditions, ACC is upregulated in most types of cancer such as liver, breast, and prostate cancer (89) and is downregulated in PTC. The downregulation of ACC2 *via* BRAF^V600E^ plays a critical role in PTC and establishes favorable conditions for TC cell proliferation (90). Of the lipogenic enzymes upregulated in ATC, stearoyl-CoA desaturase-1 (SCD1) that can mediate the desaturation of endogenously synthesized saturated fatty acids into monounsaturated fatty acids (MUFAs) and promote the proliferation of various cancer cell types showed the most significant differential expression when compared with that in normal thyroid tissues (91). A highly positive correlation between MUFAs and monounsaturated phosphatidylcholines (MUPCs) and negative correlations between monosaturated and polyunsaturated lipids have been observed in many types of cancers including breast, lung, colorectal, esophageal, and gastric cancer; thus, similar lipogenic mechanisms may exist to generate the lipids. However, it should be noted that a lower correlation than that mentioned above in TC was observed (92) (**Table 1**). These findings suggest the presence of different lipid metabolism in TC while it is not clear at this stage. Overall, these cases support the view that TC cells are dependent on *de novo* lipogenesis for cell viability (**Figure 2**).

#### Enhancement of Protein Synthesis

As a crucial component of all cells and tissues of the human body, proteins are the material basis of life. Proteins have many functions in organisms, including catalysis, locomotion, transport, mechanical support, immunity, regulation. Protein synthesis consists of five steps, including amino acid activation, initiation of polypeptide chain synthesis, peptide chain extension, peptide chain termination and release, and post-synthesis processing and modification of the protein. This process expresses the genetic information on messenger RNA (mRNA) transcribed from DNA in the form of proteins. As tumor cells are more metabolically active and divide more frequently than normal cells, they require more proteins.

As mentioned above, the PI3K-Akt-mTOR pathway is activated in various kinds of carcinoma. This pathway is also closely associated with protein synthesis. Tumor cells keep their protein synthesis positive to meet the growth needs through this pathway. In addition, tumor cells have different genetic mutations that activate the synthesis of certain proteins and perform certain functions.

Ribosomes, ribonucleoprotein particles in cells, are mainly composed of numerous distinct proteins and rRNA and are responsible for protein synthesis. In recent decades, many studies have demonstrated the causal associations between inherited mutations affecting ribosome biogenesis and increased cancer risk. Recent studies have shown that dysregulated ribosome biogenesis plays a broader role in the development and progression of most cancers (93–98). Some studies have also assessed the relationship between ribosomes and TC. Saiselet et al. reported that the expression of genes involved in the negative regulation of cell death/apoptosis was also downregulated in five TC cell lines (WRO, FTC133, BCPAP, TPC1, and K1) (99). Jeong et al. discovered the high expression of LXRβ in TC, which was coordinately associated with ribosome-related genes (100).

#### Abnormalities in Nucleic Acid Biosynthesis

Nucleic acid is a biological macromolecule with a nucleotide as its basic unit, which has a complex spatial structure and important biological functions. Nucleic acids can be classified as deoxyribonucleic acid (DNA) and ribonucleic acid (RNA). DNA, which is found in the nucleus and mitochondria, carries genetic information and is passed down through generations through replication. Cell and organismal traits are determined by this genetic information. The two basic pathways of nucleotide synthesis are *de novo* synthesis and remediation. The *de novo* synthesis of nucleotides from simple materials such as ribose phosphate, amino acids, one-carbon units, and CO_2_ is the main synthesis pathway in the human body. The *in vivo* use of free bases or nucleosides can generate nucleotides through a simple reaction process known as the salvage pathway. Tumor cells use both pathways because they require significant amounts of nucleic acids for rapid growth. As mentioned above, the catabolism of glutamine is particularly active in tumor cells; thus, increased amounts of the breakdown products of glutamine are observed when compared with those in normal cells. Ammonia produced by the breakdown of glutamine participates in the ammonia cycle and can be used for the biosynthesis of nucleotides and proteins (101–105).

Tumor cells increase nucleotide synthesis to satisfy their need for growth and proliferation (106). Therefore, the activity of nucleotide synthetase, especially deoxyribonuclease, is higher in tumor cells than that in normal cells (107). The expression of deoxyribonuclease in normal cells fluctuates with changes in the cell cycle. Cancer cells have lost normal regulation and the expression levels are constitutively high, leading to increased DNA synthesis (24). The expression levels of genes involved in DNA replication were upregulated in TC cell lines such as BCPAP and 8505C (99). The occurrence of thyroid tumors is related to abnormal nucleic acid synthesis caused by a variety of gene mutations. The activation of BRAF mutations is a major oncogenic driver of many cancers, especially TC (108, 109). BRAF is the predominant mutation (30–40%) in PTC and is considered an initiating event in papillary thyroid carcinogenesis. Another human gene involved in thyroid carcinogenesis is TERT, which contributes to the distant metastasis (110–112).

## TC Cell Metabolism and the TME

### Tumor Cell Metabolism Shapes the Inflammatory TME

The two major characteristics of the TME are hypoxia and acidification, which are closely related. Tumor cells increase glycolysis to adapt to the hypoxic microenvironment. The lactate produced by glycolysis, in turn, acidifies the TME. In addition, the incomplete vasculature of tumor tissue prevents the timely elimination of metabolites, which is also related to the acidification of the TME. Active metabolism in TME cells can also lead to increased toxic concentrations of certain metabolites, such as increased levels of adenosine, kynurenine, ornithine, reactive oxygen species, and potassium. These metabolites have profound effects on suppressing the tumor immune response. During tumor development, the TME changes continuously with tumor growth and develop its cellular contents by releasing various recruiting factors, leading to the accumulation of specific types of immune cells in the TME, also affects the functions of these immune cells and the complex relationship between these cells and tumor cells. Thus, tumors are no longer simply a problem of cancer cells. Co-evolution occurs between tumor cells and the surrounding stromal cells, forming an inseparable community. Under the influence of tumor cells, tumor stromal fibroblasts, macrophages, and neutrophils become tumor-associated fibroblasts (CAFs), tumor-associated macrophages (TAMs), and tumor-associated neutropenia.

### Metabolic Crosstalk in the TC Microenvironment

#### Nutrient Competition

The high metabolic activity of cancer cells and the disordered vasculature in the TME can contribute to a microenvironment featuring nutrient depletion and hypoxia, which established a metabolic competition between cancer cells and infiltrating immune cells. This series of changes and metabolic reprogramming plays a significant role in promoting tumor growth and immune escape. Chen et al. compared human normal thyroid and PTC samples and identified metabolites in carbohydrate metabolism, including glucose, that consistently decreased in PTC (113). The lack of glucose impaired the function of immune cells such as TAMs and T cells by regulating mTOR and GAPDH. Glycolysis promotes effector T cell (Teff cell) function by sustaining the production of IFNγ. Decreased mTOR activity diminishes IFNγ at the transcriptional level in CD8^+^ T cells and, thus, impairs T cell function (114, 115). Besides glucose, amino acids also play a role in driving and fueling T cell function and differentiation. The neighboring immune cells in solid tumors are outcompeted due to arginine uptake and catabolism which primarily shifts toward cancer cells (116). Leone et al. reported that tumor cells exposed to glutamine antagonist showed decreased viability, proliferation, and cell cycle progression while Teff cells produce a long-lived, highly activated phenotype by markedly upregulating oxidative metabolism (117).

#### Secreted Metabolites

The accumulation of metabolites such as lactate, kynurenine, and other metabolic by-products of cancer metabolism can be detrimental to immune cells, leading to tumor immunosuppression. Indoleamine 2, 3-dioxygenase (IDO), a rate-limiting enzyme in tryptophan oxidation, promotes tryptophan uptake from the TME and generates kynurenine, which inhibits tryptophan import. Therefore, the amino acids of T cells are depleted and result in immunosuppression and induced T cell apoptosis. IDO-expressing tumor cells are not rejected by specific T cells through the secretion of kynurenines, which can suppress cytotoxic effector functions *via* the downregulation of TCR CD3 ζ-chain and induced FOXP3^+^ regulatory T cell (Treg) differentiation. IDO upregulation impaired the function of NK cell function and boost the high infiltration of FOXP3^+^ Tregs in thyroid carcinoma (118, 119). In addition, Foxp3^+^ Tregs in lymphocytes facilitate thyroid tumor growth and invasion (120). A large amount of lactate can also cause acidosis in the microenvironment and weaken immune cell function (121). Arts et al. showed that TC-derived lactate-mediated TC-induced TAM reprogramming and inflammation through Akt/mTOR-dependent glycolysis, an increase in inflammation characteristics, and changes in cell metabolism (122). The accumulation of lactate is also detrimental to the function and antitumor response of T and NK cells by inhibiting proliferation and cytokine production (123). These studies suggested that patients with cancer should be cautious when using lactate preparations, as lactate may promote tumor growth.

Tumor cells also secrete vascular endothelial growth factor (VEGF) into the TME, resulting in the upregulation of 6-phosphofructo-2-kinase/fructose-2, 6-biphosphatase 3(PFKFB3) in endothelial cells, which activates PFK-1 to promote the glycolytic phenotype as well as proliferation (124). Colegio et al. demonstrated that lactate produced by tumor cells promotes M2 macrophage polarization by a HIF1a-dependent mechanism. In turn, VEGF and Arginase-1 secreted by M2-polarized macrophages signal back to tumor cells and promote tumor growth (125).

#### Metabolic Coupling

In TME, the energy metabolism of CAFs shifts to aerobic glycolysis under the influence of cancer cells. The lactate, ketone body, or pyruvate released by these CAFs can be used as an energy source by epithelial cancer cells to enter the TCA cycle and produce ATP through OXPHOS. This phenomenon is called the reverse Warburg effect. Lactate produced by CAFs is exported *via* the monocarboxylate transporter (MCT)-4 into the TME and taken up by tumor cells *via* the MCT-1 transporter. Such metabolic coupling has been reported in several tumor types including head and neck cancer (126). In addition, the metabolic coupling between PTC cells and adjacent fibroblasts can result in aggressive behavior owing to the large-scale production lactate, which is transported outside the cell by MCT4 (127). CAFs also increased the anabolic metabolism of glutamine which can be consumed by cancer cells to sustain nucleotide generation and OXPHOS. In contrast, glutamate secreted by cancer cells promoted the production of glutathione (GSH), thereby maintaining redox balance and ECM remodeling in CAFs (128). The results of Mestre-Farrera et al. indicated that glutamine deprivation promoted CAFs migration and invasion, which, in turn, promotes tumor epithelial cells to move to nutrient-rich areas (129). CAFs release paracrine signals to induce metabolic reprogramming and epigenetic changes, causing changes similar to KRAS-driven oncogenic transformations (130). Tumors cells release factors such as PDGF and TGF-β, resulting in metabolic reprogramming of CAFs toward aerobic glycolysis (131, 132). Fozzatti et al. described the significant increase of GLUT-1 in human fibroblasts *in vitro* when cultured in ATC cells-derived conditioned media. Strikingly, conditioned media obtained from these activated fibroblasts promoted cell proliferation and invasion of follicular TC cell line (133). Rabold et al. performed transcriptome, metabolome, and lipidome analyses on TC-induced macrophages in a human coculture model. The lipidome analysis showed increased total lipid and intracellular lipid content of tumor-induced macrophages, especially phosphoglycerides and sphingolipids. Remarkably, this metabolic shift in lipid synthesis contributes to their protumoral functional characteristics: a block of key enzymes of lipid biosynthesis in tumor-induced macrophages reversed elevated inflammatory cytokines and the ability to produce ROS, two well-known pro-tumoral factors in the TME (134).

**Figure 3:**
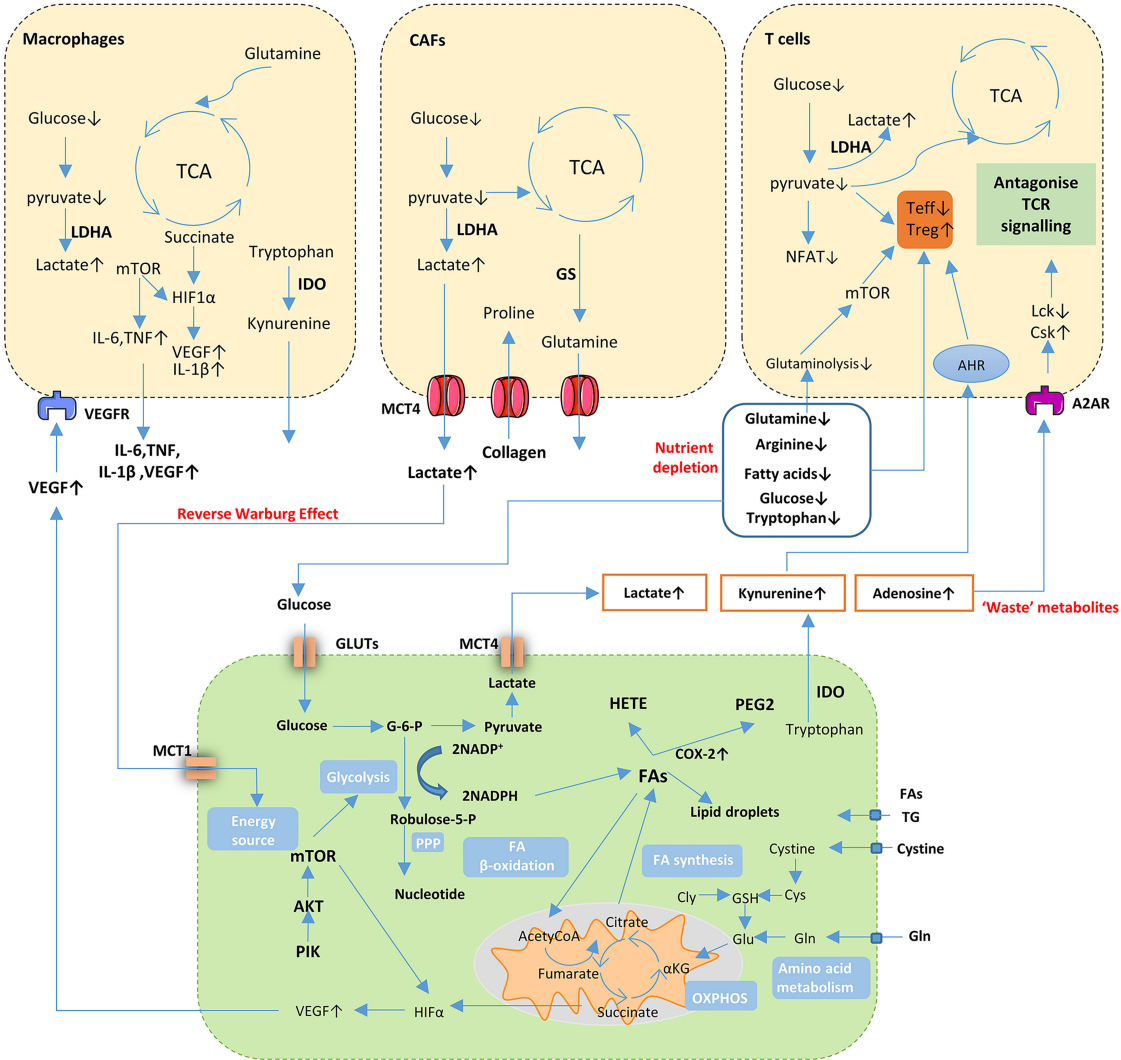
Cancer cell metabolism and crosstalk in the TME. Cancer cells undergo metabolic changes including activation of aerobic glycolytic, enhanced FA synthesis and increased uptake of glutamine supply for bioenergetics through tricarboxylic acid (TCA) cycle and support biosynthesis of proteins. Nutrient depletion, accumulation of ‘waste’ metabolites and aberrant signaling molecules in TME influence the function and proliferation of both cancer cells and immune or stromal cells. Gln, glutamine; Glu, Glutamate; Cys, cysteine; GSH, glutathione; Cly, glycine; TG, triglyceride; FA, fatty acids; PPP, pentose phosphate pathway; NADH, nicotinamide adenine dinucleotide; NADPH, nicotinamide adenine dinucleotide phosphate; HETE, thromboxane hydroxiepoxyeicosate-traenoic acid; PEG2, prostaglandin E2; COX-2, cyclooxygenases-2; G-6-P, glucose-6-phosphate; IDO, Indoleamine 2, 3-dioxygenase; MCT, monocarboxylate transporter; GLUT, glucose transporter; VEGF, vascular endothelial growth factor; LDHA, lactate dehydrogenase A; GS, glutamine synthetase; NFTA, nuclear factor of activated T cells; AHR, aryl hydrocarbon receptor; A2AR, Adenosine 2A receptor; Csk, C-terminal Src kinase; Lck, lymphocyte-specific protein tyrosine kinase; Teff, effector T cells; Treg, regulatory T cells.

These studies show the complicated and dynamic interaction that exists between thyroid tumors and immune cells in TME, which results in the promotion of thyroid tumorigenesis (**Figure 3**).

## Prognostic Biomarkers and Treatment

### Prognostic Indicators

**Figure 4:**
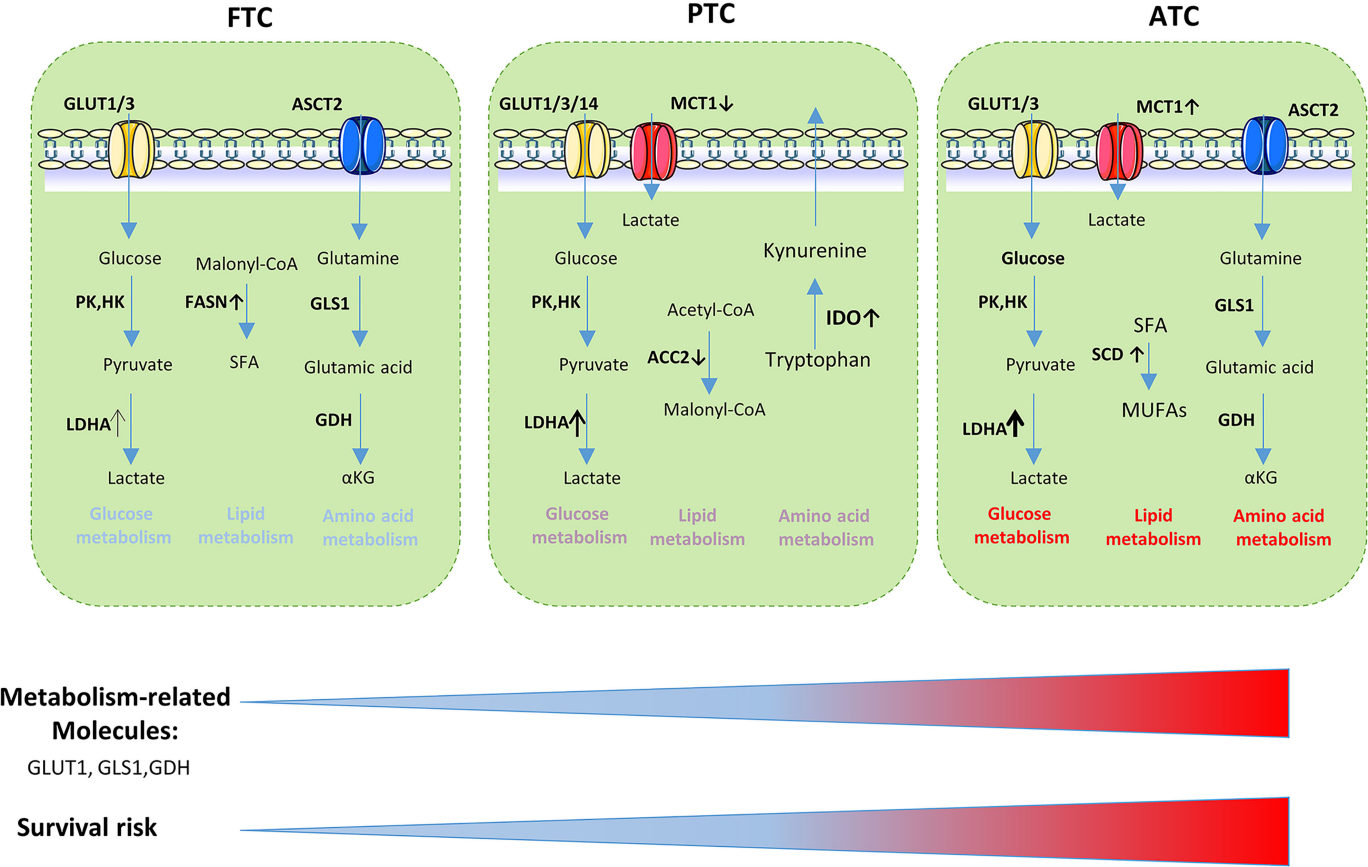
Metabolism-related molecules is related to the aggressiveness of thyroid cancer and survival risk. GLUT, glucose transporter; LDHA, lactate dehydrogenase A; PK, Pyruvate kinase; HK, hexokinase; SFA, saturated fatty acids; ASCT, amino acid transporter; FASN, fatty acid synthase; GLS1, glutaminase 1; GDH, glutamate dehydrogenase; ACC2, Acetyl-CoA carboxylase 2; IDO,Indoleamine 2, 3-dioxygenase; SCD1, stearoyl-CoA desaturase-1; MUFAs, monounsaturated fatty acids.

In conclusion, the expression of metabolism-related molecules revealed the differences in invasiveness and prognosis between different TC subtypes (**Figure 4**). Numerous studies have demonstrated the relationship between the prognosis of thyroid carcinoma and glycolysis-related proteins such as GLUT, LDHA, MCT1 (32, 135, 136). Some studies have indicated that GLUT contributed to the increased glucose uptake observed during carcinogenesis (135, 137). The differentiated extent of thyroid cancer is negatively correlated with the expression of GLUTs. Poorly differentiated types such as ATC have high expression levels of GLUT (mainly GLUT-1); in contrast, well-differentiated tumors such as FTC and PTC usually have low GLUT-1 expression levels (45, 137–140). Glutamine, serine, glycine, and other amino acid metabolism-related proteins can also be used as prognostic indicators for thyroid tumors. Stromal GDH positivity was an independent factor associated with poor prognosis. In follicular variant PTC, stromal serine hydromethyl transferase 1 expression was associated with shorter disease-free survival. The serine/glycine metabolism-related molecules phosphoglycerate dehydrogenase, glycine decarboxylase, and phosphoserine phosphatase positivity were associated with shorter overall survival (57, 58, 60, 141). IDO, which was associated with the aggressive features of papillary thyroid microcarcinoma, may disrupt antitumor immunity and contribute to tumor progression by increased infiltration of FOXP3^+^ Treg cells (142).

### Metabolism Targeted Therapy

At present, cancer therapeutic regimens face the problem of drug resistance which may associate with metabolic reprogramming in tumor. Therefore, combination therapies that target various tumor cell properties showed great potential value. Metabolic inhibitors in combination with targeted therapy or chemotherapy hold promise for increasing anticancer drug sensitivity.

#### Glucose Metabolism as a Therapeutic Target

The energy supply of tumor cells differs from that of normal cells. This unique energy supply pathway is mainly due to increased glycolytic enzyme expression and activity levels. Theoretically, inhibiting specific glycolytic metabolic enzymes with high expression levels can cut off the energy supply of tumor cells, while normal tissues are not affected. When the glycolytic pathway is inhibited, normal tissue cells can utilize fatty acid and amino acid production through alternative pathways. Some glycolytic enzymes, such as HK-II LDHA, and PKM2, are highly expressed in malignant tumors. These highly expressed glycolytic enzymes can be used as targets for tumor treatment (143). Due to tumor cell heterogeneity and TME variability, the expression and activity of glycolytic enzymes may change. Consequently, the therapeutic effect of a single glycolytic enzyme target may not be as good as that for the combination of multiple glycolytic enzyme targets. Combinations involving the inhibition of glycolysis and OXPHOS, or glycolysis and glutaminolysis have been proven in multiple preclinical cancer models to effectively suppress tumor growth (144–148). Glyoxalase I (GLO I) is a rate-limiting enzyme that is involved in the detoxification of cytotoxic methylglyoxal formed in glycolysis. The combination of GLO I inhibitor with shikonin, a PKM2 specific inhibitor, could suppress the cellular proliferation and induction of apoptosis (149).

Various HK2 inhibitors have been identified, including 2-deoxyglucose(2-DG), 3-bromopyruvate (3-BP), and lonidamine (LND). In thyroid tumors, glycolytic inhibitors also show unique therapeutic effects. Glycolytic inhibition with 3-BP suppress tumor growth and extends survival in a murine model of ATC when combined with the ketogenic diet (150). It has been previously shown that glycolytic inhibitors 2DG significantly enhanced the antitumor effects of other medical treatments and radiotherapy (151–154). Phase I/II clinical trials have been performed for 2-DG as a single-agent therapy in solid tumors and hormone-refractory prostate cancer. However, further research was halted owing to the significant toxicities and limited efficacy (NCT00633087) (155). LND also reached phase II and III clinical trials for the treatment of several tumor types but showed only modest clinical activity and a lack of specificity. Moreover, due to concerns regarding liver enzyme abnormalities, further research was halted (156, 157). Targeted therapy is a common treatment for thyroid tumors. When blocking platelet-derived growth factor receptor by imatinib, the pro-oncogene BRAF^V600E^ promotes thyroid tumor cell glycolysis *via* the upregulation of HK2 expression, resulting in drug resistance. However, glucose uptake and metabolism in thyroid tumor cells were downregulated when BRAF^V600E^ was blocked by vemurafenib. In terms of tumor growth, combination therapy of imatinib and vemurafenib was much more effective than single therapy and led to a near abolition of the tumors (158). The combination of imatinib and HK2 inhibitors may solve the problem of drug resistance and also provide better efficacy in TC.

LDH is a critical metabolic enzyme that is considered a hallmark of aggressive malignancies. Radiotherapy is a common therapy in thyroid cancer, indicating the combination therapy of LDHA inhibitor and radiotherapy may be efficient in thyroid cancer. Chen et al. find LDHA suppression monotherapy decreased cellular proliferation and stunted tumor growth temporarily in ATC but cannot achieve tumor cure, due to the maintenance of residual viable cells. Only the combination therapy of chronic LDHA suppression and radiation can achieve a functional cure (159). Various LDHA inhibitors have been developed, such as dichloroacetate (DCA), gossypol, oxamate and FX-11 (160–162). The lactate transporter MCT links intracellular lactate with the TME and plays an indispensable role in tumor lactate metabolism. AZD3965 is an inhibitor of the MCT-1/MTC-2 lactate transporter and reached phase I clinical trials for both solid tumors and large B-cell lymphoma (NCT01791595). However, MCT inhibition also impairs T cell proliferation (**Table 2**).

**Table 2:** Metabolism-targeting cancer therapies.

Target pathway and protein		Agent	Study phase	Effects	Interventions	References	Status
**Glucose metabolism**	HK2	2-DG	Phase II	Limited efficacy on tumor growth and significant toxicities	Single agent	NCT00633087	Terminated
		LND	Phase III	Limited efficacy and produced more myalgias and fatigue	Combined with epirubicin	(150)	
		3-BP	Preclinical	Suppresses tumor growth and improves survival *in vivo*	combined with the ketogenic diet	(143)	
	MTC1	AZD3965	Phase I		Single agent	NCT01791595	Completed
	LDHA	DCA	Phase I		Single agent	NCT01163487	Completed
		Gossypol	Phase I/II	Safe and well tolerated but shown limited activity.	Single agent	(1^153^)	
		Oxamate	Preclinical	Inhibits the viability of cancer cells in a dose- and time-dependent manner		(155)	
		FX-11	Preclinical	Block aerobic glycolysis and growth cancer *in vitro*	Single agent	(154)	
**Amino acid metabolism**	GL1	CB-839	Phase II		Combined with Paclitaxel	NCT03057600	Completed
	IDO	Epacadostat	Phase III	Effect remains uncertain.	Combined with Pembrolizumab	NCT02752074	Completed
		Indoximod	Phase II		Combined with Chemoradiotherapy	NCT04049669	Recruiting
**Lipid metabolism**	ACC	ND-654	Preclinical	Inhibits the tumor development *in vivo*, improve survival rate	Single agent; combined with the sorafenib	(68)	
	SCD	SSI-4	Preclinical	Regulate tumor-initiating cells and sorafenib resistance	Combined with sorafenib	(182)	
		Betulinic acid	Preclinical	Induces rapid cell death	Single agent	(184)	
		MF-438	Preclinical	Achieve better control	Combined with cisplatin	(183)	

#### Amino Acid Metabolism as a Therapeutic Target

Amino acids are an essential component of tumor cells and are closely related to tumor development. Thus, amino acid metabolism may provide a new therapeutic perspective. L­asparaginase is approved by the Food and Drug Administration for the front­line treatment of acute lymphoblastic leukemia (163). Other treatments for amino acid deprivation have also shown encouraging results in clinical trials in several solid malignancies (164–167). The mitochondrial enzyme GLS plays a crucial role in glutaminolysis. Among the GLS inhibitors, CB-839 is more potent, selective and shows greater bioavailability. In phase I clinical trials, CB-839 showed preliminary signs of clinical activity with an acceptable safety profile in multiple tumor types including triple-negative breast cancer, non-small cell lung adenocarcinoma, renal cell carcinoma, mesothelioma, and tumors with mutations in enzymes in the TCA cycle (NCT02071862) (168).

Since tumor cells require glutamine, one possible strategy is to treat tumors by preventing or interfering with glutamine metabolism by tumor cells. The blockade of glutamine in tumor-bearing mice inhibited cancer cell oxidation and glycolytic metabolism, resulting in hypoxia, acidosis, and reduced nutrient consumption (117). However, some studies showed that increasing the intake of glutamine in tumor-bearing rats did not elevate the growth rate of tumors; moreover, clinical work has also shown that glutamine supplementation in patients with tumors improved chemotherapy efficacy and reduced the adverse reactions (169–173). IDO, the rate­limiting enzyme in tryptophan catabolism, is highly expressed in TC cells and suppresses the function of NK cells. IDO inhibitors such as epacadostat have reached phase III trials and show promising efficacy in combination therapies by linking metabolism and immunomodulation. Therefore, IDO inhibitors are likely to be useful for the treatment of thyroid tumors (174).

#### Lipid Metabolism as a Therapeutic Target

ACC is a rate-limiting enzyme for *de novo* lipid synthesis and inhibition of fatty acid oxidation. Rescue of ACC2 may be a new molecular strategy to overcome the resistance of refractory PTC to BRAF^V600E^ inhibitors (90). SCD is an aliphatic acyl desaturase that catalyzes the transformation of saturated fatty acids into MUFAs by inserting cis-double bonds at the Δ9 position of the carbon chain (175). MUFAs play a role in cell growth, survival, differentiation, metabolic regulation, and signal transduction. SCD has been observed in a wide range of cancer cells (176–179) and this increase is closely associated with cancer aggressiveness and poor prognosis (180–183). Previous research established SCD reduces cell proliferation and invasion by blocking cell migration and membrane fluidity (184–187). In ATC, therapeutic and genetic-targeted inhibition of SCD enzyme activity promoted a significant reduction in cell proliferation and induced cell death, while normal thyroid cells were unaffected (91). SCD inhibitors such as SSI-4, betulinic acid, and MF-438 that proved effective in antitumor effect (188–190) may show a promising efficiency in the treatment of thyroid cancer.

## Conclusion and Perspective

The crucial of metabolic reprogramming in tumor development and metastasis is increasingly recognized (**Table 3**). The complicated relationship between tumor cell metabolism and the TME is also important. Tumor cell metabolism can cause acidification of the TME and can also recruit immune cells to change immune cell metabolism in the TME. However, the immune microenvironment can also act on tumor cells to promote the immune escape of tumor cells.

**Table 3:** Metabolic reprogramming between proliferation and metastasis in thyroid cancer.

Metabolism pathways		Function	Reference	Evidence
**Glucose metabolism**	LDHA	Migration, invasion, tumor growth	(26)	*In vivo and in vitro*
	HK2	Proliferation, migration	(41)	*In vitro*
**Amino acid metabolism**	IDO	Tumor growth and invasion	(135)	Clinical relevance
**Lipid metabolism**	SREBP1, SCD, FASN and ACC	Extrathyroidal extension, lymph node metastasis, migration and invasion	(61)	Clinical relevance, *in vitro*
	SCD1	Proliferation and viability	(84)	*In vitro*

Although there has been some progress in the study of metabolic reprogramming of TC in recent years, there remain many gaps to fill. Some outstanding questions still need to be addressed for the development of specific metabolic targeted therapy. More studies are needed to determine how thyroid tumor cell metabolism interacts with immune cells in the microenvironment, which metabolic targets can be blocked specifically for TC treatment, the possible side effects of metabolism inhibitors, and the solutions to these challenges.

## Author Contributions

Conceived the work: LB and ZP. Wrote the manuscript: LB. Generated data for figures: TX and XL. Revised the manuscript: MG and PH. All authors contributed to the article and approved the submitted version.

## Funding

This work was funded by the National Natural Science Foundation, People’s Republic of China (Nos. 82173157, U20A20382, 81872170, 81802673), Natural Science Foundation of Zhejiang Provincial under Grant No.Y22H168220, Chinese Medicine Research Program of Zhejiang Province (No. 2021ZA006), and Zhejiang Medical and Health Science and Technology Project (Nos. 2021KY056 and 2022KY042).

## Conflict of Interest

The authors declare that the research was conducted in the absence of any commercial or financial relationships that could be construed as a potential conflict of interest.

## Publisher’s Note

All claims expressed in this article are solely those of the authors and do not necessarily represent those of their affiliated organizations, or those of the publisher, the editors and the reviewers. Any product that may be evaluated in this article, or claim that may be made by its manufacturer, is not guaranteed or endorsed by the publisher.

## References

[1] Kim J Gosnell JE Roman SA. Geographic Influences in the Global Rise of Thyroid Cancer. Nat Rev Endocrinol (2020) 16:17–29. doi: 10.1038/s41574-019-0263-x 31616074

[2] Kitahara CM Sosa JA. The Changing Incidence of Thyroid Cancer. Nat Rev Endocrinol (2016) 12:646–53. doi: 10.1038/nrendo.2016.110 PMC10311569 27418023

[3] Cabanillas ME McFadden DG Durante C. Thyroid Cancer. Lancet (2016) 388:2783–95. doi: 10.1016/s0140-6736(16)30172-6 27240885

[4] Nikiforova MN Nikiforov YE. Molecular Genetics of Thyroid Cancer: Implications for Diagnosis, Treatment and Prognosis. Expert Rev Mol Diagn (2008) 8:83–95. doi: 10.1586/14737159.8.1.83 18088233

[5] Molinaro E Romei C Biagini A Sabini E Agate L Mazzeo S. Anaplastic Thyroid Carcinoma: From Clinicopathology to Genetics and Advanced Therapies. Nat Rev Endocrinol (2017) 13:644–60. doi: 10.1038/nrendo.2017.76 28707679

[6] Filetti S Durante C Hartl D Leboulleux S Locati LD Newbold K. Thyroid Cancer: ESMO Clinical Practice Guidelines for Diagnosis, Treatment and Follow-Up†. Ann Oncol (2019) 30:1856–83. doi: 10.1093/annonc/mdz400 31549998

[7] Grani G Lamartina L Durante C Filetti S Cooper DS. Follicular Thyroid Cancer and Hürthle Cell Carcinoma: Challenges in Diagnosis, Treatment, and Clinical Management. Lancet Diabetes Endocrinol (2018) 6:500–14. doi: 10.1016/s2213-8587(17)30325-x 29102432

[8] Tuttle RM. Controversial Issues in Thyroid Cancer Management. J Nucl Med (2018) 59:1187–94. doi: 10.2967/jnumed.117.192559 PMC6071505 29653980

[9] Raue F Frank-Raue K. Thyroid Cancer: Risk-Stratified Management and Individualized Therapy. Clin Cancer Res (2016) 22:5012–21. doi: 10.1158/1078-0432.Ccr-16-0484 27742787

[10] Hay N. Reprogramming Glucose Metabolism in Cancer: Can it be Exploited for Cancer Therapy? Nat Rev Cancer (2016) 16:635–49. doi: 10.1038/nrc.2016.77 PMC5516800 27634447

[11] Faubert B Solmonson A DeBerardinis RJ. Metabolic Reprogramming and Cancer Progression. Science (2020) 368:eaaw5473. doi: 10.1126/science.aaw5473 32273439 PMC7227780

[12] Cha YH Yook JI Kim HS Kim NH. Catabolic Metabolism During Cancer EMT. Arch Pharm Res (2015) 38:313–20. doi: 10.1007/s12272-015-0567-x 25634102

[13] Herst PM Grasso C Berridge MV. Metabolic Reprogramming of Mitochondrial Respiration in Metastatic Cancer. Cancer Metastasis Rev (2018) 37:643–53. doi: 10.1007/s10555-018-9769-2 30448881

[14] Zhao H Li Y. Cancer Metabolism and Intervention Therapy. Mol Biomedicine (2021) 2:5. doi: 10.1186/s43556-020-00012-1 PMC8607959 35006438

[15] Yang Z Huang R Wei X Yu W Min Z Ye M. The SIRT6-Autophagy-Warburg Effect Axis in Papillary Thyroid Cancer. Front Oncol (2020) 10:1265. doi: 10.3389/fonc.2020.01265 32983963 PMC7485319

[16] Chen J Zhou Q Feng J Zheng W Du J Meng X. Activation of AMPK Promotes Thyroid Cancer Cell Migration Through Its Interaction With PKM2 and β-Catenin. Life Sci (2019) 239:116877. doi: 10.1016/j.lfs.2019.116877 31669575

[17] Wang R Cheng Y Su D Gong B He X Zhou X. Cpt1c Regulated by AMPK Promotes Papillary Thyroid Carcinomas Cells Survival Under Metabolic Stress Conditions. J Cancer (2017) 239:3675–81. doi: 10.7150/jca.21148 PMC5688920 29151954

[18] Kishore M Cheung KCP Fu H Bonacina F Wang G Coe D. Regulatory T Cell Migration Is Dependent on Glucokinase-Mediated Glycolysis. Immunity (2017) 47:875–89.e10. doi: 10.1016/j.immuni.2017.10.017 29166588 PMC5714502

[19] Li L Liang Y Kang L Liu Y Gao S Chen S. Transcriptional Regulation of the Warburg Effect in Cancer by SIX1. Cancer Cell (2018) 33:368–85.e7. doi: 10.1016/j.ccell.2018.01.010 29455928

[20] Hensley CT Faubert B Yuan Q Lev-Cohain N Jin E Kim J. Metabolic Heterogeneity in Human Lung Tumors. CELL (2016) 164:681–94. doi: 10.1016/j.cell.2015.12.034 PMC4752889 26853473

[21] Christen S Lorendeau D Schmieder R Broekaert D Metzger K Veys K. Breast Cancer-Derived Lung Metastases Show Increased Pyruvate Carboxylase-Dependent Anaplerosis. Cell Rep (2016) 17:837–48. doi: 10.1016/j.celrep.2016.09.042 27732858

[22] Chen J Lee HJ Wu X Huo L Kim SJ Xu L. Gain of Glucose-Independent Growth Upon Metastasis of Breast Cancer Cells to the Brain. Cancer Res (2015) 75:554–65. doi: 10.1158/0008-5472.Can-14-2268 PMC4315743 25511375

[23] Mulvey PF Jr. Kelleher JJ Slingerland DW. Oxidation of Glucose-C14 by Human Thyroid Tissues. Metabolism (1963) 12:829–32. 14062766

[24] Hengstschläger M Mudrak I Wintersberger E Wawra E. A Common Regulation of Genes Encoding Enzymes of the Deoxynucleotide Metabolism is Lost After Neoplastic Transformation. Cell Growth Differ (1994) 5:1389–94. 7696188

[25] Gao Y Yang F Yang XA Zhang L Yu H Cheng X. Mitochondrial Metabolism is Inhibited by the HIF1α-MYC-PGC-1β Axis in BRAF V600E Thyroid Cancer. FEBS J (2019) 286:1420–36. doi: 10.1111/febs.14786 30768848

[26] Zhou L Cha G Chen L Yang C Xu D Ge M. HIF1α/PD-L1 Axis Mediates Hypoxia-Induced Cell Apoptosis and Tumor Progression in Follicular Thyroid Carcinoma. Onco Targets Ther (2019) 12:6461–70. doi: 10.2147/ott.S203724 PMC6698605 31616157

[27] Yang Z Yu W Huang R Ye M Min Z. SIRT6/HIF-1α Axis Promotes Papillary Thyroid Cancer Progression by Inducing Epithelial-Mesenchymal Transition. Cancer Cell Int (2019) 19:17. doi: 10.1186/s12935-019-0730-4 30675128 PMC6335740

[28] Klaus A Fathi O Tatjana TW Bruno N Oskar K. Expression of Hypoxia-Associated Protein HIF-1α in Follicular Thyroid Cancer is Associated With Distant Metastasis. Pathol Oncol Res (2018) 24:289–96. doi: 10.1007/s12253-017-0232-4 28474313

[29] Yu L Lu M Jia D Ma J Ben-Jacob E Levine H. Modeling the Genetic Regulation of Cancer Metabolism: Interplay Between Glycolysis and Oxidative Phosphorylation. Cancer Res (2017) 77:1564–74. doi: 10.1158/0008-5472.Can-16-2074 PMC5380541 28202516

[30] Matijevic Glavan T Cipak Gasparovic A Vérillaud B Busson P Pavelic J. Toll-Like Receptor 3 Stimulation Triggers Metabolic Reprogramming in Pharyngeal Cancer Cell Line Through Myc, MAPK, and HIF. Mol Carcinog (2017) 56:1214–26. doi: 10.1002/mc.22584 27805282

[31] Choudhry H Harris AL. Advances in Hypoxia-Inducible Factor Biology. Cell Metab (2018) 27:281–98. doi: 10.1016/j.cmet.2017.10.005 29129785

[32] Hou X Shi X Zhang W Li D Hu L Yang J. LDHA Induces EMT Gene Transcription and Regulates Autophagy to Promote the Metastasis and Tumorigenesis of Papillary Thyroid Carcinoma. Cell Death Dis (2021) 12:347. doi: 10.1038/s41419-021-03641-8 33795650 PMC8017009

[33] Jin L Chun J Pan C Alesi GN Li D Magliocca KR. Phosphorylation-Mediated Activation of LDHA Promotes Cancer Cell Invasion and Tumour Metastasis. Oncogene (2017) 36:3797–806. doi: 10.1038/onc.2017.6 PMC5501759 28218905

[34] Matsuzu K Segade F Matsuzu U Carter A Bowden DW Perrier ND. Differential Expression of Glucose Transporters in Normal and Pathologic Thyroid Tissue. Thyroid (2004) 14:806–12. doi: 10.1089/thy.2004.14.806 15588375

[35] Grabellus F Nagarajah J Bockisch A Schmid KW Sheu SY. Glucose Transporter 1 Expression, Tumor Proliferation, and Iodine/Glucose Uptake in Thyroid Cancer With Emphasis on Poorly Differentiated Thyroid Carcinoma. Clin Nucl Med (2012) 35:121–7. doi: 10.1097/RLU.0b013e3182393599 22228332

[36] Ciampi R Vivaldi A Romei C Del Guerra A Salvadori P Cosci B. Expression Analysis of Facilitative Glucose Transporters (Gluts) in Human Thyroid Carcinoma Cell Lines and Primary Tumors. Mol Cell Endocrinol (2008) 291:57–62. doi: 10.1016/j.mce.2008.05.003 18571834

[37] Samih N Hovsepian S Aouani A Lombardo D Fayet G. Glut-1 Translocation in FRTL-5 Thyroid Cells: Role of Phosphatidylinositol 3-Kinase and N-Glycosylation. Endocrinology (2000) 141:4146–55. doi: 10.1210/endo.141.11.7793 11089547

[38] Szablewski L. Expression of Glucose Transporters in Cancers. Biochim Biophys Acta (2013) 1835:164–9. doi: 10.1016/j.bbcan.2012.12.004 23266512

[39] Jóźwiak P Krześlak A Pomorski L Lipińska A. Expression of Hypoxia-Related Glucose Transporters GLUT1 and GLUT3 in Benign, Malignant and Non-Neoplastic Thyroid Lesions. Mol Med Rep (2012) 6:601–6. doi: 10.3892/mmr.2012.969 22752218

[40] Chai YJ Yi JW Oh SW Kim YA Yi KH Kim JH. Upregulation of SLC2 (GLUT) Family Genes is Related to Poor Survival Outcomes in Papillary Thyroid Carcinoma: Analysis of Data From the Cancer Genome Atlas. Surgery (2017) 12:188–94. doi: 10.1016/j.surg.2016.04.050 27842912

[41] Valli A Morotti M Zois CE Albers PK Soga T Feldinger K. Adaptation to HIF1α Deletion in Hypoxic Cancer Cells by Upregulation of GLUT14 and Creatine Metabolism. Mol Cancer Res (2019) 17:1531–44. doi: 10.1158/1541-7786.Mcr-18-0315 30885992

[42] Haber RS Weiser KR Pritsker A Reder I Burstein DE. GLUT1 Glucose Transporter Expression in Benign and Malignant Thyroid Nodules. Thyroid (1997) 7:363–7. doi: 10.1089/thy.1997.7.363 9226204

[43] Xue C Li G Bao Z Zhou Z Li L. Mitochondrial Pyruvate Carrier 1: A Novel Prognostic Biomarker That Predicts Favourable Patient Survival in Cancer. Cancer Cell Int (2021) 21:288. doi: 10.1186/s12935-021-01996-8 34059057 PMC8166087

[44] Cairns RA Harris IS Mak TW. Regulation of Cancer Cell Metabolism. Nat Rev Cancer (2011) 11:85–95. doi: 10.1038/nrc2981 21258394

[45] Nahm JH Kim HM Koo JS. Glycolysis-Related Protein Expression in Thyroid Cancer. Tumour Biol (2017) 39:1010428317695922. doi: 10.1177/1010428317695922 28347233

[46] Hooft L van der Veldt AA Hoekstra OS Boers M Molthoff CF van Diest PJ. Hexokinase III, Cyclin a and Galectin-3 are Overexpressed in Malignant Follicular Thyroid Nodules. Clin Endocrinol (Oxf) (2008) 68:252–7. doi: 10.1111/j.1365-2265.2007.03031.x 17868400

[47] Huang J Gao W Liu H Yin G Duan H Huang Z. Up-Regulated ANP32E Promotes the Thyroid Carcinoma Cell Proliferation and Migration *via* Activating AKT/Mtor/HK2-Mediated Glycolysis. Gene (2020) 750:144681. doi: 10.1016/j.gene.2020.144681 32304784

[48] Feng C Gao Y Wang C Yu X Zhang W Guan H. Aberrant Overexpression of Pyruvate Kinase M2 Is Associated With Aggressive Tumor Features and the BRAF Mutation in Papillary Thyroid Cancer. J Clin Endocrinol Metab (2013) 98:E1524–33. doi: 10.1210/jc.2012-4258 23846818

[49] Strickaert A Corbet C Spinette SA Craciun L Dom G Andry G. Reprogramming of Energy Metabolism: Increased Expression and Roles of Pyruvate Carboxylase in Papillary Thyroid Cancer. Thyroid (2019) 29:845–57. doi: 10.1089/thy.2018.0435 30990120

[50] Verhagen JN van der Heijden MC de Jong-van Dijken J Rijksen G der Kinderen PJ van Unnik JA. Pyruvate Kinase in Normal Human Thyroid Tissue and Thyroid Neoplasms. Cancer (1985) 55:142–8. doi: 10.1002/1097-0142(19850101)55:1<142::aid-cncr2820550122>3.0.co;2-x 3965076

[51] Hosios AM Hecht VC Danai LV Johnson MO Rathmell JC Steinhauser ML. Amino Acids Rather Than Glucose Account for the Majority of Cell Mass in Proliferating Mammalian Cells. Dev Cell (2016) 36:540–9. doi: 10.1016/j.devcel.2016.02.012 PMC4766004 26954548

[52] Zhang J Pavlova NN Thompson CB. Cancer Cell Metabolism: The Essential Role of the Nonessential Amino Acid, Glutamine. EMBO J (2017) 36:1302–15. doi: 10.15252/embj.201696151 PMC5430235 28420743

[53] Kodama M Oshikawa K Shimizu H Yoshioka S Takahashi M Izumi Y. A Shift in Glutamine Nitrogen Metabolism Contributes to the Malignant Progression of Cancer. Nat Commun (2020) 11:1320. doi: 10.1038/s41467-020-15136-9 32184390 PMC7078194

[54] Altman BJ Stine ZE Dang CV. From Krebs to Clinic: Glutamine Metabolism to Cancer Therapy. Nat Rev Cancer (2016) 16:619–34. doi: 10.1038/nrc.2016.71 PMC5484415 27492215

[55] Cluntun AA Lukey MJ Cerione RA Locasale JW. Glutamine Metabolism in Cancer: Understanding the Heterogeneity. Trends Cancer (2017) 3:169–80. doi: 10.1016/j.trecan.2017.01.005 PMC5383348 28393116

[56] Chen Z Lin J Feng S Chen X Huang H Wang C. SIRT4 Inhibits the Proliferation, Migration, and Invasion Abilities of Thyroid Cancer Cells by Inhibiting Glutamine Metabolism. Onco Targets Ther (2019) 12:2397–408. doi: 10.2147/ott.S189536 PMC6445187 30992675

[57] Kim HM Lee YK Koo JS. Expression of Glutamine Metabolism-Related Proteins in Thyroid Cancer. Oncotarget (2016) 7:53628–41. doi: 10.18632/oncotarget.10682 PMC5288210 27447554

[58] Cha YJ Jang H Koo JS. Expression of Glutamine Metabolism-Related Proteins in Hürthle Cell Neoplasm of Thyroid: Comparison With Follicular Neoplasm. Histol Histopathol (2019) 34:167–74. doi: 10.14670/hh-18-036 30191947

[59] Gebregiworgis T Purohit V Shukla SK Tadros S Chaika NV Abrego J. Glucose Limitation Alters Glutamine Metabolism in MUC1-Overexpressing Pancreatic Cancer Cells. J Proteome Res (2017) 16:3536–46. doi: 10.1021/acs.jproteome.7b00246 PMC5634392 28809118

[60] Sun WY Kim HM Jung WH Koo JS. Expression of Serine/Glycine Metabolism-Related Proteins is Different According to the Thyroid Cancer Subtype. J Transl Med (2016) 14:168. doi: 10.1186/s12967-016-0915-8 27277113 PMC4898323

[61] Zhao M Bu Y Feng J Zhang H Chen Y Yang G. SPIN1 Triggers Abnormal Lipid Metabolism and Enhances Tumor Growth in Liver Cancer. Cancer Lett (2020) 470:54–63. doi: 10.1016/j.canlet.2019.11.032 31790762

[62] Yi M Li J Chen S Cai J Ban Y Peng Q. Emerging Role of Lipid Metabolism Alterations in Cancer Stem Cells. J Exp Clin Cancer Res (2018) 16:118. doi: 10.1186/s13046-018-0784-5 PMC6003041 29907133

[63] Röhrig F Schulze A. The Multifaceted Roles of Fatty Acid Synthesis in Cancer. Nat Rev Cancer (2016) 16:732–49. doi: 10.1038/nrc.2016.89 27658529

[64] Mossmann D Park S Hall MN. Mtor Signalling and Cellular Metabolism are Mutual Determinants in Cancer. Nat Rev Cancer (2018) 18:744–57. doi: 10.1038/s41568-018-0074-8 30425336

[65] Luo X Cheng C Tan Z Li N Tang M Yang L. Emerging Roles of Lipid Metabolism in Cancer Metastasis. Mol Cancer (2017) 16:76. doi: 10.1186/s12943-017-0646-3 28399876 PMC5387196

[66] Li H Feng Z He ML. Lipid Metabolism Alteration Contributes to and Maintains the Properties of Cancer Stem Cells. Theranostics (2020) 10:7053–69. doi: 10.7150/thno.41388 PMC7330842 32641978

[67] Cheng C Geng F Cheng X Guo D. Lipid Metabolism Reprogramming and its Potential Targets in Cancer. Cancer Commun (Lond) (2018) 38:27. doi: 10.1186/s40880-018-0301-4 29784041 PMC5993136

[68] Liao T Wang YJ Hu JQ Wang Y Han LT Ma B. Histone Methyltransferase KMT5A Gene Modulates Oncogenesis and Lipid Metabolism of Papillary Thyroid Cancer *In Vitro* . Oncol Rep (2018) 39:2185–92. doi: 10.3892/or.2018.6295 29512765

[69] Liu H Liu JY Wu X Zhang JT. Biochemistry, Molecular Biology, and Pharmacology of Fatty Acid Synthase, an Emerging Therapeutic Target and Diagnosis/Prognosis Marker. Int J Biochem Mol Biol (2010) 1:69–89. 20706604 PMC2919769

[70] Fantin VR St-Pierre J Leder P. Attenuation of LDH-a Expression Uncovers a Link Between Glycolysis, Mitochondrial Physiology, and Tumor Maintenance. Cancer Cell (2006) 9:425–34. doi: 10.1016/j.ccr.2006.04.023 16766262

[71] Zhang C Liu J Huang G Zhao Y Yue X Wu H. Cullin3-KLHL25 Ubiquitin Ligase Targets ACLY for Degradation to Inhibit Lipid Synthesis and Tumor Progression. Genes Dev (2016) 30:1956–70. doi: 10.1101/gad.283283.116 PMC5066239 27664236

[72] Svensson RU Parker SJ Eichner LJ Kolar MJ Wallace M Brun SN. Inhibition of Acetyl-Coa Carboxylase Suppresses Fatty Acid Synthesis and Tumor Growth of Non-Small-Cell Lung Cancer in Preclinical Models. Nat Med (2016) 22:1108–19. doi: 10.1038/nm.4181 PMC5053891 27643638

[73] Rios Garcia M Steinbauer B Srivastava K Singhal M Mattijssen F Maida A. Acetyl-Coa Carboxylase 1-Dependent Protein Acetylation Controls Breast Cancer Metastasis and Recurrence. Cell Metab (2017) 26:842–55.e5. doi: 10.1016/j.cmet.2017.09.018 29056512

[74] Nwosu ZC Battello N Rothley M Piorońska W Sitek B Ebert MP. Liver Cancer Cell Lines Distinctly Mimic the Metabolic Gene Expression Pattern of the Corresponding Human Tumours. J Exp Clin Cancer Res (2018) 37:211. doi: 10.1186/s13046-018-0872-6 30176945 PMC6122702

[75] Lally JSV Ghoshal S DePeralta DK Moaven O Wei L Masia R. Inhibition of Acetyl-Coa Carboxylase by Phosphorylation or the Inhibitor ND-654 Suppresses Lipogenesis and Hepatocellular Carcinoma. Cell Metab (2019) 29:174–82.e5. doi: 10.1016/j.cmet.2018.08.020 30244972 PMC6643297

[76] Kapadia B Nanaji NM Bhalla K Bhandary B Lapidus R Beheshti A. Fatty Acid Synthase Induced S6Kinase Facilitates USP11-Eif4b Complex Formation for Sustained Oncogenic Translation in DLBCL. Nat Commun (2018) 9:829. doi: 10.1038/s41467-018-03028-y 29483509 PMC5827760

[77] Gu L Zhu Y Lin X Lu B Zhou X Zhou F. The Ikkβ-USP30-ACLY Axis Controls Lipogenesis and Tumorigenesis. Hepatology (2021) 73:160–74. doi: 10.1002/hep.31249 32221968

[78] Gouw AM Margulis K Liu NS Raman SJ Mancuso A Toal GG. The MYC Oncogene Cooperates With Sterol-Regulated Element-Binding Protein to Regulate Lipogenesis Essential for Neoplastic Growth. Cell Metab (2019) 30:556–72.e5. doi: 10.1016/j.cmet.2019.07.012 31447321 PMC6911354

[79] Corbet C Pinto A Martherus R Santiago de Jesus JP Polet F Feron O. Acidosis Drives the Reprogramming of Fatty Acid Metabolism in Cancer Cells Through Changes in Mitochondrial and Histone Acetylation. Cell Metab (2016) 24:311–23. doi: 10.1016/j.cmet.2016.07.003 27508876

[80] Che L Chi W Qiao Y Zhang J Song X Liu Y. Cholesterol Biosynthesis Supports the Growth of Hepatocarcinoma Lesions Depleted of Fatty Acid Synthase in Mice and Humans. Gut (2020) 69:177–86. doi: 10.1136/gutjnl-2018-317581 PMC6943247 30954949

[81] Bort A Sánchez BG de Miguel I Mateos-Gómez PA Diaz-Laviada I. Dysregulated Lipid Metabolism in Hepatocellular Carcinoma Cancer Stem Cells. Mol Biol Rep (2020) 47:2635–47. doi: 10.1007/s11033-020-05352-3 32125560

[82] Ali A Levantini E Teo JT Goggi J Clohessy JG Wu CS. Fatty Acid Synthase Mediates EGFR Palmitoylation in EGFR Mutated Non-Small Cell Lung Cancer. EMBO Mol Med (2018) 10. doi: 10.15252/emmm.201708313 PMC5840543 29449326

[83] Abbassi-Ghadi N Antonowicz SS McKenzie JS Kumar S Huang J Jones EA. De Novo Lipogenesis Alters the Phospholipidome of Esophageal Adenocarcinoma. Cancer Res (2020) 80:2764–74. doi: 10.1158/0008-5472.Can-19-4035 32345674

[84] Xu M Sun T Wen S Zhang T Wang X Cao Y. Characteristics of Lipid Metabolism-Related Gene Expression-Based Molecular Subtype in Papillary Thyroid Cancer. Acta Biochim Biophys Sin (Shanghai) (2020) 52:1166–70. doi: 10.1093/abbs/gmaa092 32880619

[85] Leng J Guan Q Sun T Wu Y Cao Y Guo Y. Application of Isotope-Based Carboxy Group Derivatization in LC-MS/MS Analysis of Tissue Free-Fatty Acids for Thyroid Carcinoma. J Pharm BioMed Anal (2013) 84:256–62. doi: 10.1016/j.jpba.2013.06.004 23867087

[86] Uddin S Siraj AK Al-Rasheed M Ahmed M Bu R Myers JN. Fatty Acid Synthase and AKT Pathway Signaling in a Subset of Papillary Thyroid Cancers. J Clin Endocrinol Metab (2008) 93:4088–97. doi: 10.1210/jc.2008-0503 18682509

[87] Sekiguchi M Shiroko Y Arai T Kishino T Sugawara I Kusakabe T. Biological Characteristics and Chemosensitivity Profile of Four Human Anaplastic Thyroid Carcinoma Cell Lines. BioMed Pharmacother (2001) 55:466–74. doi: 10.1016/s0753-3322(01)00087-7 11686581

[88] Liu J Brown RE. Immunohistochemical Expressions of Fatty Acid Synthase and Phosphorylated C-Met in Thyroid Carcinomas of Follicular Origin. Int J Clin Exp Pathol (2011) 4:755–64. PMC3225787 22135723

[89] Munir R Lisec J Swinnen JV Zaidi N. Lipid Metabolism in Cancer Cells Under Metabolic Stress. Br J Cancer (2019) 120:1090–8. doi: 10.1038/s41416-019-0451-4 PMC6738079 31092908

[90] Valvo V Iesato A Kavanagh TR Priolo C Zsengeller Z Pontecorvi A. Fine-Tuning Lipid Metabolism by Targeting Mitochondria-Associated Acetyl-Coa-Carboxylase 2 in BRAF(V600E) Papillary Thyroid Carcinoma. Thyroid (2021) 31:1335–58. doi: 10.1089/thy.2020.0311 PMC8558082 33107403

[91] von Roemeling CA Marlow LA Pinkerton AB Crist A Miller J Tun HW. Aberrant Lipid Me{Von Roemeling, 2015 #353}Tabolism in Anaplastic Thyroid Carcinoma Reveals Stearoyl Coa Desaturase 1 as a Novel Therapeutic Target. J Clin Endocrinol Metab (2015) 100:E697–709. doi: 10.1210/jc.2014-2764 PMC4422887 25675381

[92] Guo S Wang Y Zhou D Li Z. Significantly Increased Monounsaturated Lipids Relative to Polyunsaturated Lipids in Six Types of Cancer Microenvironment are Observed by Mass Spectrometry Imaging. Sci Rep (2014) 4:5959. doi: 10.1038/srep05959 25091112 PMC4121604

[93] Turi Z Lacey M Mistrik M Moudry P. Impaired Ribosome Biogenesis: Mechanisms and Relevance to Cancer and Aging. Aging (Albany NY) (2019) 11:2512–40. doi: 10.18632/aging.101922 PMC6520011 31026227

[94] Prakash V Carson BB Feenstra JM Dass RA Sekyrova P Hoshino A. Ribosome Biogenesis During Cell Cycle Arrest Fuels EMT in Development and Disease. Nat Commun (2019) 10:2110. doi: 10.1038/s41467-019-10100-8 31068593 PMC6506521

[95] Penzo M Montanaro L Treré D Derenzini M. The Ribosome Biogenesis-Cancer Connection. Cells (2019) 8. doi: 10.3390/cells8010055 PMC6356843 30650663

[96] Pelletier J Thomas G Volarević S. Ribosome Biogenesis in Cancer: New Players and Therapeutic Avenues. Nat Rev Cancer (2018) 18:51–63. doi: 10.1038/nrc.2017.104 29192214

[97] Kim DS Camacho CV Nagari A Malladi VS Challa S Kraus WL. Activation of PARP-1 by Snornas Controls Ribosome Biogenesis and Cell Growth via the RNA Helicase DDX21. Mol Cell (2019) 75:1270–85.e14. doi: 10.1016/j.molcel.2019.06.020 31351877 PMC6754283

[98] Catez F Dalla Venezia N Marcel V Zorbas C Lafontaine DLJ Diaz JJ. Ribosome Biogenesis: An Emerging Druggable Pathway for Cancer Therapeutics. Biochem Pharmacol (2019) 175:74–81. doi: 10.1016/j.bcp.2018.11.014 30468711

[99] Saiselet M Floor S Tarabichi M Dom G Hébrant A van Staveren WC. Thyroid Cancer Cell Lines: An Overview. Front Endocrinol (Lausanne) (2012) 3:133. doi: 10.3389/fendo.2012.00133 23162534 PMC3499787

[100] Jeong S Kim IK Kim H Choi MJ Lee J Jo YS. Liver X Receptor β Related to Tumor Progression and Ribosome Gene Expression in Papillary Thyroid Cancer. Endocrinol Metab (Seoul) (2020) 35:656–68. doi: 10.3803/EnM.2020.667 PMC7520597 32814418

[101] Wang Y Bai C Ruan Y Liu M Chu Q Qiu L. Coordinative Metabolism of Glutamine Carbon and Nitrogen in Proliferating Cancer Cells Under Hypoxia. Nat Commun (2019) 10:201. doi: 10.1038/s41467-018-08033-9 30643150 PMC6331631

[102] Tardito S Oudin A Ahmed SU Fack F Keunen O Zheng L. Glutamine Synthetase Activity Fuels Nucleotide Biosynthesis and Supports Growth of Glutamine-Restricted Glioblastoma. Nat Cell Biol (2015) 17:1556–68. doi: 10.1038/ncb3272 PMC4663685 26595383

[103] Kodama M Nakayama KI. A Second Warburg-Like Effect in Cancer Metabolism: The Metabolic Shift of Glutamine-Derived Nitrogen: A Shift in Glutamine-Derived Nitrogen Metabolism From Glutaminolysis to *De Novo* Nucleotide Biosynthesis Contributes to Malignant Evolution of Cancer. BIOESSAYS (2020) 42:e2000169. doi: 10.1002/bies.202000169 33165972

[104] Fu S Li Z Xiao L Hu W Zhang L Xie B. Glutamine Synthetase Promotes Radiation Resistance via Facilitating Nucleotide Metabolism and Subsequent DNA Damage Repair. Cell Rep (2019) 28:1136–43.e4. doi: 10.1016/j.celrep.2019.07.002 31365859

[105] Bott AJ Shen J Tonelli C Zhan L Sivaram N Jiang YP. Glutamine Anabolism Plays a Critical Role in Pancreatic Cancer by Coupling Carbon and Nitrogen Metabolism. Cell Rep (2019) 29:1287–98.e6. doi: 10.1016/j.celrep.2019.09.056 31665640 PMC6886125

[106] Lv Y Wang X Li X Xu G Bai Y Wu J. Nucleotide *De Novo* Synthesis Increases Breast Cancer Stemness and Metastasis *via* Cgmp-PKG-MAPK Signaling Pathway. PloS Biol (2020) 18:e3000872. doi: 10.1371/journal.pbio.3000872 33186350 PMC7688141

[107] Chan EM Shibue T McFarland JM Gaeta B Ghandi M Dumont N. WRN Helicase is a Synthetic Lethal Target in Microsatellite Unstable Cancers. NATURE (2019) 568:551–6. doi: 10.1038/s41586-019-1102-x PMC6580861 30971823

[108] Robb R Yang L Shen C Wolfe AR Webb A Zhang X. Inhibiting BRAF Oncogene-Mediated Radioresistance Effectively Radiosensitizes BRAF(V600E)-Mutant Thyroid Cancer Cells by Constraining DNA Double-Strand Break Repair. Clin Cancer Res (2019) 25:4749–60. doi: 10.1158/1078-0432.Ccr-18-3625 PMC6677585 31097454

[109] Cabanillas ME Ferrarotto R Garden AS Ahmed S Busaidy NL Dadu R. Neoadjuvant BRAF- and Immune-Directed Therapy for Anaplastic Thyroid Carcinoma. THYROID (2018) 28:945–51. doi: 10.1089/thy.2018.0060 PMC6425982 29742974

[110] Trybek T Walczyk A Gąsior-Perczak D Pałyga I Mikina E Kowalik A. Impact of BRAF V600E and TERT Promoter Mutations on Response to Therapy in Papillary Thyroid Cancer. Endcrinology (2019) 160:2328–38. doi: 10.1210/en.2019-00315 31305897

[111] Panebianco F Nikitski AV Nikiforova MN Nikiforov YE. Spectrum of TERT Promoter Mutations and Mechanisms of Activation in Thyroid Cancer. Cancer Med (2019) 8:5831–9. doi: 10.1002/cam4.2467 PMC6792496 31408918

[112] Melo M Gaspar da Rocha A Batista R Vinagre J Martins MJ Costa G. TERT, BRAF, and NRAS in Primary Thyroid Cancer and Metastatic Disease. J Clin Endocrinol Metab (2017) 102:1898–907. doi: 10.1210/jc.2016-2785 28323937

[113] Chen M Shen M Li Y Liu C Zhou K Hu W. GC-MS-Based Metabolomic Analysis of Human Papillary Thyroid Carcinoma Tissue. Int J Mol Med (2015) 36:1607–14. doi: 10.3892/ijmm.2015.2368 26459747

[114] Cham CM Gajewski TF. Glucose Availability Regulates IFN-Gamma Production and P70s6 Kinase Activation in CD8+ Effector T Cells. J Immunol (2005) 174:4670–7. doi: 10.4049/jimmunol.174.8.4670 15814691

[115] Chang CH Curtis JD Maggi LB Jr. Faubert B Villarino AV O’Sullivan D. Posttranscriptional Control of T Cell Effector Function by Aerobic Glycolysis. CELL (2013) 153:1239–51. doi: 10.1016/j.cell.2013.05.016 PMC3804311 23746840

[116] Elia I Haigis MC. Metabolites and the Tumour Microenvironment: From Cellular Mechanisms to Systemic Metabolism. Nat Metab (2021) 3:21–32. doi: 10.1038/s42255-020-00317-z 33398194 PMC8097259

[117] Leone RD Zhao L Englert JM Sun IM Oh MH Sun IH. Glutamine Blockade Induces Divergent Metabolic Programs to Overcome Tumor Immune Evasion. Science (2019) 366:1013–21. doi: 10.1126/science.aav2588 PMC7023461 31699883

[118] Moretti S Menicali E Voce P Morelli S Cantarelli S Sponziello M. Indoleamine 2,3-Dioxygenase 1 (IDO1) is Up-Regulated in Thyroid Carcinoma and Drives the Development of an Immunosuppressant Tumor Microenvironment. J Clin Endocrinol Metab (2014) 99:E832–40. doi: 10.1210/jc.2013-3351 24517146

[119] Park A Yang Y Lee Y Kim MS Park YJ Jung H. Indoleamine-2,3-Dioxygenase in Thyroid Cancer Cells Suppresses Natural Killer Cell Function by Inhibiting NKG2D and Nkp46 Expression *via* STAT Signaling Pathways. J Clin Med (2019) 8. doi: 10.3390/jcm8060842 PMC6617210 31212870

[120] Chu R Liu SY Vlantis AC van Hasselt CA Ng EK Fan MD. Inhibition of Foxp3 in Cancer Cells Induces Apoptosis of Thyroid Cancer Cells. Mol Cell Endocrinol (2015) 399:228–34. doi: 10.1016/j.mce.2014.10.006 25312920

[121] Ivashkiv LB. The Hypoxia-Lactate Axis Tempers Inflammation. Nat Rev Immunol (2020) 20:85–6. doi: 10.1038/s41577-019-0259-8 PMC7021227 31819164

[122] Arts RJ Plantinga TS Tuit S Ulas T Heinhuis B Tesselaar M. Transcriptional and Metabolic Reprogramming Induce an Inflammatory Phenotype in Non-Medullary Thyroid Carcinoma-Induced Macrophages. OncoImmunology (2016) 5:e1229725. doi: 10.1080/2162402x.2016.1229725 28123869 PMC5213309

[123] Brand A Singer K Koehl GE Kolitzus M Schoenhammer G Thiel A. LDHA-Associated Lactic Acid Production Blunts Tumor Immunosurveillance by T and NK Cells. Cell Metab (2016) 24:657–71. doi: 10.1016/j.cmet.2016.08.011 27641098

[124] Cantelmo AR Conradi LC Brajic A Goveia J Kalucka J Pircher A. Inhibition of the Glycolytic Activator PFKFB3 in Endothelium Induces Tumor Vessel Normalization, Impairs Metastasis, and Improves Chemotherapy. Cancer Cell (2016) 30:968–85. doi: 10.1016/j.ccell.2016.10.006 PMC5675554 27866851

[125] Colegio OR Chu NQ Szabo AL Chu T Rhebergen AM Jairam V. Functional Polarization of Tumour-Associated Macrophages by Tumour-Derived Lactic Acid. Nature (2014) 513:559–63. doi: 10.1038/nature13490 PMC4301845 25043024

[126] Curry JM Tuluc M Whitaker-Menezes D Ames JA Anantharaman A Butera A. Cancer Metabolism, Stemness and Tumor Recurrence: MCT1 and MCT4 are Functional Biomarkers of Metabolic Symbiosis in Head and Neck Cancer. Cell Cycle (2013) 12:1371–84. doi: 10.4161/cc.24092 PMC3674065 23574725

[127] Curry JM Tassone P Cotzia P Sprandio J Luginbuhl A Cognetti DM. Multicompartment Metabolism in Papillary Thyroid Cancer. Laryngoscope (2016) 126:2410–8. doi: 10.1002/lary.25799 PMC4909595 26666958

[128] Bertero T Oldham WM Grasset EM Bourget I Boulter E Pisano S. Tumor-Stroma Mechanics Coordinate Amino Acid Availability to Sustain Tumor Growth and Malignancy. Cell Metab (2019) 29:124–40.e10. doi: 10.1016/j.cmet.2018.09.012 30293773 PMC6432652

[129] Mestre-Farrera A Bruch-Oms M Peña R Rodríguez-Morató J Alba-Castellón L Comerma L. Glutamine-Directed Migration of Cancer-Activated Fibroblasts Facilitates Epithelial Tumor Invasion. Cancer Res (2021) 81:438–51. doi: 10.1158/0008-5472.Can-20-0622 33229340

[130] Sherman MH Yu RT Tseng TW Sousa CM Liu S Truitt ML. Stromal Cues Regulate the Pancreatic Cancer Epigenome and Metabolome. Proc Natl Acad Sci USA (2017) 114:1129–34. doi: 10.1073/pnas.1620164114 PMC5293019 28096419

[131] Cadamuro M Brivio S Mertens J Vismara M Moncsek A Milani C. Platelet-Derived Growth Factor-D Enables Liver Myofibroblasts to Promote Tumor Lymphangiogenesis in Cholangiocarcinoma. J Hepatol (2019) 70:700–9. doi: 10.1016/j.jhep.2018.12.004 PMC10878126 30553841

[132] Cruz-Bermúdez A Laza-Briviesca R Vicente-Blanco RJ García-Grande A Coronado MJ Laine-Menéndez S. Cancer-Associated Fibroblasts Modify Lung Cancer Metabolism Involving ROS and TGF-β Signaling. Free Radic Biol Med (2019) 130:163–73. doi: 10.1016/j.freeradbiomed.2018.10.450 30391585

[133] Fozzatti L Alamino VA Park S Giusiano L Volpini X Zhao L. Interplay of Fibroblasts With Anaplastic Tumor Cells Promotes Follicular Thyroid Cancer Progression. Sci Rep (2019) 9:8028. doi: 10.1038/s41598-019-44361-6 31142771 PMC6541589

[134] Rabold K Aschenbrenner A Thiele C Boahen CK Schiltmans A Smit JWA. Enhanced Lipid Biosynthesis in Human Tumor-Induced Macrophages Contributes to Their Protumoral Characteristics. J Immunother Cancer (2020) 8. doi: 10.1136/jitc-2020-000638 PMC7500191 32943450

[135] Schönberger J Rüschoff J Grimm D Marienhagen J Rümmele P Meyringer R. Glucose Transporter 1 Gene Expression is Related to Thyroid Neoplasms With an Unfavorable Prognosis: An Immunohistochemical Study. THYROID (2002) 12:747–54. doi: 10.1089/105072502760339307 12481939

[136] Johnson JM Lai SY Cotzia P Cognetti D Luginbuhl A Pribitkin EA. Mitochondrial Metabolism as a Treatment Target in Anaplastic Thyroid Cancer. Semin Oncol (2015) 42:915–22. doi: 10.1053/j.seminoncol.2015.09.025 PMC4663018 26615136

[137] Matsuzu K Segade F Wong M Clark OH Perrier ND Bowden DW. Glucose Transporters in the Thyroid. Thyroid (2005) 15:545–50. doi: 10.1089/thy.2005.15.545 16029120

[138] Yang H Zhong JT Zhou SH Han HM. Roles of GLUT-1 and HK-II Expression in the Biological Behavior of Head and Neck Cancer. Oncotarget (2019) 10:3066–83. doi: 10.18632/oncotarget.24684 PMC6508962 31105886

[139] Suh HY Choi H Paeng JC Cheon GJ Chung JK Kang KW. Comprehensive Gene Expression Analysis for Exploring the Association Between Glucose Metabolism and Differentiation of Thyroid Cancer. BMC Cancer (2019) 19:1260. doi: 10.1186/s12885-019-6482-7 31888560 PMC6937781

[140] Kim S Chung JK Min HS Kang JH Park DJ Jeong JM. Expression Patterns of Glucose Transporter-1 Gene and Thyroid Specific Genes in Human Papillary Thyroid Carcinoma. Nucl Med Mol Imaging (2014) 48:91–7. doi: 10.1007/s13139-013-0249-x PMC4028475 24900148

[141] Huang FQ Li J Jiang L Wang FX Alolga RN Wang MJ. Serum-Plasma Matched Metabolomics for Comprehensive Characterization of Benign Thyroid Nodule and Papillary Thyroid Carcinoma. Int J Cancer (2019) 144:868–76. doi: 10.1002/ijc.31925 30318614

[142] Ryu HS Park YS Park HJ Chung YR Yom CK Ahn SH. Expression of Indoleamine 2,3-Dioxygenase and Infiltration of FOXP3+ Regulatory T Cells are Associated With Aggressive Features of Papillary Thyroid Microcarcinoma. Thyroid (2014) 42:1232–40. doi: 10.1089/thy.2013.0423 24742251

[143] Hsieh IS Gopula B Chou CC Wu HY Chang GD Wu WJ. Development of Novel Irreversible Pyruvate Kinase M2 Inhibitors. J Med Chem (2019) 62:8497–510. doi: 10.1021/acs.jmedchem.9b00763 31465224

[144] Vangapandu HV Alston B Morse J Ayres ML Wierda WG Keating MJ. Biological and Metabolic Effects of IACS-010759, an Oxphos Inhibitor, on Chronic Lymphocytic Leukemia Cells. Oncotarget (2018) 9:24980–91. doi: 10.18632/oncotarget.25166 PMC5982765 29861847

[145] Bizjak M Malavašič P Dolinar K Pohar J Pirkmajer S Pavlin M. Combined Treatment With Metformin and 2-Deoxy Glucose Induces Detachment of Viable MDA-MB-231 Breast Cancer Cells. vitro Sci Rep (2017) 7:1761. doi: 10.1038/s41598-017-01801-5 28496098 PMC5431940

[146] Jones AT Narov K Yang J Sampson JR Shen MH. Efficacy of Dual Inhibition of Glycolysis and Glutaminolysis for Therapy of Renal Lesions in Tsc2(+/-) Mice. Neoplasia (2019) 21:230–8. doi: 10.1016/j.neo.2018.12.003 PMC6324218 30622053

[147] Sun Y Bandi M Lofton T Smith M Bristow CA Carugo A. Functional Genomics Reveals Synthetic Lethality Between Phosphogluconate Dehydrogenase and Oxidative Phosphorylation. Cell Rep (2019) 26:469–82.e5. doi: 10.1016/j.celrep.2018.12.043 30625329

[148] DeWaal D Nogueira V Terry AR Patra KC Jeon SM Guzman G. Hexokinase-2 Depletion Inhibits Glycolysis and Induces Oxidative Phosphorylation in Hepatocellular Carcinoma and Sensitizes to Metformin. Nat Commun (2018) 9:446. doi: 10.1038/s41467-017-02733-4 29386513 PMC5792493

[149] Shimada N Takasawa R Tanuma SI. Interdependence of GLO I and PKM2 in the Metabolic Shift to Escape Apoptosis in GLO I-Dependent Cancer Cells. Arch Biochem Biophys (2018) 638:1–7. doi: 10.1016/j.abb.2017.12.008 29225125

[150] Zhao B Aggarwal A Marshall JA Barletta JA Kijewski MF Lorch JH. Glycolytic Inhibition With 3-Bromopyruvate Suppresses Tumor Growth and Improves Survival in a Murine Model of Anaplastic Thyroid Cancer. Surgery (2021). doi: 10.1016/j.surg.2021.05.055 34334212

[151] Wang SY Wei YH Shieh DB Lin LL Cheng SP Wang PW. 2-Deoxy-D-Glucose can Complement Doxorubicin and Sorafenib to Suppress the Growth of Papillary Thyroid Carcinoma Cells. PloS One (2015) 10:e0130959. doi: 10.1371/journal.pone.0130959 26134286 PMC4489888

[152] Sobhakumari A Orcutt KP Love-Homan L Kowalski CE Parsons AD Knudson CM. 2-Deoxy-D-Glucose Suppresses the in Vivo Antitumor Efficacy of Erlotinib in Head and Neck Squamous Cell Carcinoma Cells. Oncol Res (2016) 24:55–64. doi: 10.3727/096504016x14586627440192 27178822 PMC5282972

[153] Sandulache VC Skinner HD Wang Y Chen Y Dodge CT Ow TJ. Glycolytic Inhibition Alters Anaplastic Thyroid Carcinoma Tumor Metabolism and Improves Response to Conventional Chemotherapy and Radiation. Mol Cancer Ther (2012) 11:1373–80. doi: 10.1158/1535-7163.Mct-12-0041 PMC3856684 22572813

[154] Robbins RJ Wan Q Grewal RK Reibke R Gonen M Strauss HW. Real-Time Prognosis for Metastatic Thyroid Carcinoma Based on 2-[18F]Fluoro-2-Deoxy-D-Glucose-Positron Emission Tomography Scanning. J Clin Endocrinol Metab (2006) 91:498–505. doi: 10.1210/jc.2005-1534 16303836

[155] Li J Eu JQ Kong LR Wang L Lim YC Goh BC. Targeting Metabolism in Cancer Cells and the Tumour Microenvironment for Cancer Therapy. Molecules (2020) 25. doi: 10.3390/molecules25204831 PMC7588013 33092283

[156] Nancolas B Guo L Zhou R Nath K Nelson DS Leeper DB. The Anti-Tumour Agent Lonidamine is a Potent Inhibitor of the Mitochondrial Pyruvate Carrier and Plasma Membrane Monocarboxylate Transporters. Biochem J (2016) 473:929–36. doi: 10.1042/bj20151120 PMC4814305 26831515

[157] Berruti A Bitossi R Gorzegno G Bottini A Alquati P De Matteis A. Time to Progression in Metastatic Breast Cancer Patients Treated With Epirubicin is Not Improved by the Addition of Either Cisplatin or Lonidamine: Final Results of a Phase III Study With a Factorial Design. J Clin Oncol (2002) 20:4150–9. doi: 10.1200/jco.2002.08.012 12377958

[158] Wagner M Wuest M Lopez-Campistrous A Glubrecht D Dufour J Jans HS. Tyrosine Kinase Inhibitor Therapy and Metabolic Remodelling in Papillary Thyroid Cancer. Endocr Relat Cancer (2020) 27:495–507. doi: 10.1530/erc-20-0135 32590338

[159] Chen Y Maniakas A Tan L Cui M Le X Niedzielski JS. Development of a Rational Strategy for Integration of Lactate Dehydrogenase a Suppression Into Therapeutic Algorithms for Head and Neck Cancer. Br J Cancer (2021) 124:1670–9. doi: 10.1038/s41416-021-01297-x PMC8110762 33742144

[160] Van Poznak C Seidman AD Reidenberg MM Moasser MM Sklarin N Van Zee K. Oral Gossypol in the Treatment of Patients With Refractory Metastatic Breast Cancer: A Phase I/II Clinical Trial. Breast Cancer Res Treat (2001) 66:239–48. doi: 10.1023/a:1010686204736 11510695

[161] Rellinger EJ Craig BT Alvarez AL Dusek HL Kim KW Qiao J. FX11 Inhibits Aerobic Glycolysis and Growth of Neuroblastoma Cells. SURGERY (2017) 161:747–52. doi: 10.1016/j.surg.2016.09.009 PMC5369647 27919448

[162] Zhao Z Han F Yang S Wu J Zhan W. Oxamate-Mediated Inhibition of Lactate Dehydrogenase Induces Protective Autophagy in Gastric Cancer Cells: Involvement of the Akt-Mtor Signaling Pathway. Cancer Lett (2015) 358:17–26. doi: 10.1016/j.canlet.2014.11.046 25524555

[163] Pieters R Appel I Kuehnel HJ Tetzlaff-Fohr I Pichlmeier U van der Vaart I. Pharmacokinetics, Pharmacodynamics, Efficacy, and Safety of a New Recombinant Asparaginase Preparation in Children With Previously Untreated Acute Lymphoblastic Leukemia: A Randomized Phase 2 Clinical Trial. Blood (2008) 112:4832–8. doi: 10.1182/blood-2008-04-149443 18805963

[164] Zhai L Spranger S Binder DC Gritsina G Lauing KL Giles FJ. Molecular Pathways: Targeting IDO1 and Other Tryptophan Dioxygenases for Cancer Immunotherapy. Clin Cancer Res (2015) 21:5427–33. doi: 10.1158/1078-0432.Ccr-15-0420 PMC4681601 26519060

[165] Joyce JA Fearon DT. T Cell Exclusion, Immune Privilege, and the Tumor Microenvironment. SCIENCE (2015) 348:74–80. doi: 10.1126/science.aaa6204 25838376

[166] Glazer ES Piccirillo M Albino V Di Giacomo R Palaia R Mastro AA. Phase II Study of Pegylated Arginine Deiminase for Nonresectable and Metastatic Hepatocellular Carcinoma. J Clin Oncol (2010) 28:2220–6. doi: 10.1200/jco.2009.26.7765 20351325

[167] Ascierto PA Scala S Castello G Daponte A Simeone E Ottaiano A. Pegylated Arginine Deiminase Treatment of Patients With Metastatic Melanoma: Results From Phase I and II Studies. J Clin Oncol (2005) 23:7660–8. doi: 10.1200/jco.2005.02.0933 16234528

[168] Harding JJ Telli ML Munster PN Le MH Molineaux C Bennett MK. Safety and Tolerability of Increasing Doses of CB-839, a First-in-Class, Orally Administered Small Molecule Inhibitor of Glutaminase, in Solid Tumors. J OF Clin Oncol (2015) 33:2512–2. doi: 10.1200/jco.2015.33.15_suppl.2512

[169] Wu JM Ho TW Lai IR Chen CN Lin MT. Parenteral Glutamine Supplementation Improves Serum Albumin Values in Surgical Cancer Patients. Clin Nutr (2021) 40:645–50. doi: 10.1016/j.clnu.2020.06.015 32713723

[170] Sands S Ladas EJ Kelly KM Weiner M Lin M Ndao DH. Glutamine for the Treatment of Vincristine-Induced Neuropathy in Children and Adolescents With Cancer. Support Care Cancer (2017) 25:701–8. doi: 10.1007/s00520-016-3441-6 PMC5598352 27830395

[171] Azman M Mohd Yunus MR Sulaiman S Syed Omar SN. Enteral Glutamine Supplementation in Surgical Patients With Head and Neck Malignancy: A Randomized Controlled Trial. Head Neck (2015) 37:1799–807. doi: 10.1002/hed.23839 24992652

[172] Anderson PM Lalla RV. Glutamine for Amelioration of Radiation and Chemotherapy Associated Mucositis During Cancer Therapy. Nutrients (2020) 12. doi: 10.3390/nu12061675 PMC7352314 32512833

[173] Abe T Hosoi T Kawai R Uemura N Higaki E An B. Perioperative Enteral Supplementation With Glutamine, Fiber, and Oligosaccharide Reduces Early Postoperative Surgical Stress Following Esophagectomy for Esophageal Cancer. Esophagus (2019) 16:63–70. doi: 10.1007/s10388-018-0630-z 30030739

[174] Long GV Dummer R Hamid O Gajewski TF Caglevic C Dalle S. Epacadostat Plus Pembrolizumab Versus Placebo Plus Pembrolizumab in Patients With Unresectable or Metastatic Melanoma (ECHO-301/KEYNOTE-252): A Phase 3, Randomised, Double-Blind Study. Lancet Oncol (2019) 20:1083–97. doi: 10.1016/s1470-2045(19)30274-8 31221619

[175] Ntambi JM. Regulation of Stearoyl-Coa Desaturase by Polyunsaturated Fatty Acids and Cholesterol. J Lipid Res (1999) 1549–58. doi: 10.1016/S0022-2275(20)33401-5 10484602

[176] Presler M Wojtczyk-Miaskowska A Schlichtholz B Kaluzny A Matuszewski M Mika A. Increased Expression of the Gene Encoding Stearoyl-Coa Desaturase 1 in Human Bladder Cancer. Mol Cell Biochem (2018) 40:217–24. doi: 10.1007/s11010-018-3306-z PMC6133071 29396722

[177] Lai KKY Kweon SM Chi F Hwang E Kabe Y Higashiyama R. Stearoyl-Coa Desaturase Promotes Liver Fibrosis and Tumor Development in Mice via a Wnt Positive-Signaling Loop by Stabilization of Low-Density Lipoprotein-Receptor-Related Proteins 5 and 6. Gastroenterology (2017) 447:1477–91. doi: 10.1053/j.gastro.2017.01.021 PMC5406249 28143772

[178] Gao Y Li J Xi H Cui J Zhang K Zhang J. Stearoyl-Coa-Desaturase-1 Regulates Gastric Cancer Stem-Like Properties and Promotes Tumour Metastasis *via* Hippo/YAP Pathway. Br J Cancer (2020) 152:1837–47. doi: 10.1038/s41416-020-0827-5 PMC7283337 32350414

[179] Aljohani A Khan MI Bonneville A Guo C Jeffery J O’Neill L. Hepatic Stearoyl Coa Desaturase 1 Deficiency Increases Glucose Uptake in Adipose Tissue Partially Through the PGC-1α-FGF21 Axis in Mice. J Biol Chem (2019) 122:19475–85. doi: 10.1074/jbc.RA119.009868 PMC6926457 31690632

[180] Wang J Xu Y Zhu L Zou Y Kong W Dong B. High Expression of Stearoyl-Coa Desaturase 1 Predicts Poor Prognosis in Patients With Clear-Cell Renal Cell Carcinoma. PloS One (2016) 294:e0166231. doi: 10.1371/journal.pone.0166231 PMC5115711 27861513

[181] Peck B Schulze A. Lipid Desaturation - the Next Step in Targeting Lipogenesis in Cancer? FEBS J (2016) 11:2767–78. doi: 10.1111/febs.13681 26881388

[182] Liu G Feng S Jia L Wang C Fu Y Luo Y. Lung Fibroblasts Promote Metastatic Colonization Through Upregulation of Stearoyl-Coa Desaturase 1 in Tumor Cells. Oncogene (2018) 283:1519–33. doi: 10.1038/s41388-017-0062-6 29326439

[183] Gao J Zhang Z Liu Y Zhang Z Wang M Gong A. Stearoyl-Coa Desaturase 1 Potentiates Hypoxic Plus Nutrient-Deprived Pancreatic Cancer Cell Ferroptosis Resistance. Oxid Med Cell Longev (2021) 37:6629804. doi: 10.1155/2021/6629804 PMC8032529 33868572

[184] Tutino V Gigante I Scavo MP Refolo MG Nunzio V Milella RA. Stearoyl-Coa Desaturase-1 Enzyme Inhibition by Grape Skin Extracts Affects Membrane Fluidity in Human Colon Cancer Cell Lines. Nutrients (2020) 2021:6629804. doi: 10.3390/nu12030693 PMC7146266 32143529

[185] Pisanu ME Maugeri-Saccà M Fattore L Bruschini S De Vitis C Tabbì E. Inhibition of Stearoyl-Coa Desaturase 1 Reverts BRAF and MEK Inhibition-Induced Selection of Cancer Stem Cells in BRAF-Mutated Melanoma. J Exp Clin Cancer Res (2018) 12:318. doi: 10.1186/s13046-018-0989-7 PMC6298024 30558661

[186] Piao C Cui X Zhan B Li J Li Z Li Z. Inhibition of Stearoyl Coa Desaturase-1 Activity Suppresses Tumour Progression and Improves Prognosis in Human Bladder Cancer. J Cell Mol Med (2019) 37:2064–76. doi: 10.1111/jcmm.14114 PMC6378218 30592142

[187] Ma XL Sun YF Wang BL Shen MN Zhou Y Chen JW. Sphere-Forming Culture Enriches Liver Cancer Stem Cells and Reveals Stearoyl-Coa Desaturase 1 as a Potential Therapeutic Target. BMC Cancer (2019) 23:760. doi: 10.1186/s12885-019-5963-z PMC6676608 31370822

[188] Ma MKF Lau EYT Leung DHW Lo J Ho NPY Cheng LKW. Stearoyl-Coa Desaturase Regulates Sorafenib Resistance via Modulation of ER Stress-Induced Differentiation. J Hepatol (2017) 19:979–90. doi: 10.1016/j.jhep.2017.06.015 28647567

[189] Pisanu ME Noto A De Vitis C Morrone S Scognamiglio G Botti G. Blockade of Stearoyl-Coa-Desaturase 1 Activity Reverts Resistance to Cisplatin in Lung Cancer Stem Cells. Cancer Lett (2017) 67:93–104. doi: 10.1016/j.canlet.2017.07.027 28797843

[190] Potze L di Franco S Kessler JH Stassi G Medema JP. Betulinic Acid Kills Colon Cancer Stem Cells. Curr Stem Cell Res Ther (2016) 11:427–33. doi: 10.2174/1574888x11666151203223512 26647913

